# Genome of the sea anemone *Exaiptasia pallida* and transcriptome profiles during tentacle regeneration

**DOI:** 10.3389/fcell.2022.900321

**Published:** 2022-08-17

**Authors:** Cheryl W.Y. Shum, Wenyan Nong, Wai Lok So, Yiqian Li, Zhe Qu, Ho Yin Yip, Thomas Swale, Put O. Ang, King Ming Chan, Ting Fung Chan, Ka Hou Chu, Apple P.Y. Chui, Kwok Fai Lau, Sai Ming Ngai, Fei Xu, Jerome H.L. Hui

**Affiliations:** ^1^ School of Life Sciences, Simon F.S. Li Marine Science Laboratory, State Key Laboratory of Agrobiotechnology, The Chinese University of Hong Kong, Hong Kong, China; ^2^ Dovetail Genomics, Scotts Valley, CA, United States; ^3^ Institute of Space and Earth Information Science, The Chinese University of Hong Kong, Shatin, Hong Kong SAR, China; ^4^ School of Life Sciences, The Chinese University of Hong Kong, Hong Kong, China; ^5^ School of Life Sciences, State Key Laboratory of Agrobiotechnology, The Chinese University of Hong Kong, Hong Kong, China; ^6^ Southern Marine Science and Engineering Guangdong Laboratory (Guangzhou), Guangzhou, China; ^7^ CAS and Shandong Province Key Laboratory of Experimental Marine Biology, Center for Ocean Mega-Science, Institute of Oceanology, Chinese Academy of Sciences, Qingdao, China

**Keywords:** cnidarian, sea anemone, genome, regeneration, transcriptome, microRNA

## Abstract

Cnidarians including sea anemones, corals, hydra, and jellyfishes are a group of animals well known for their regeneration capacity. However, how non-coding RNAs such as microRNAs (also known as miRNAs) contribute to cnidarian tissue regeneration is poorly understood. Here, we sequenced and assembled the genome of the sea anemone *Exaiptasia pallida* collected in Hong Kong waters. The assembled genome size of *E. pallida* is 229.21 Mb with a scaffold N50 of 10.58 Mb and BUSCO completeness of 91.1%, representing a significantly improved genome assembly of this species. The organization of ANTP-class homeobox genes in this anthozoan further supported the previous findings in jellyfishes, where most of these genes are mainly located on three scaffolds. Tentacles of *E. pallida* were excised, and both mRNA and miRNA were sequenced at 9 time points (0 h, 6 h, 12 h, 18 h, 1 day, 2, 3, 6, and 8 days) from regenerating tentacles. In addition to the Wnt signaling pathway and homeobox genes that are shown to be likely involved in tissue regeneration as in other cnidarians, we have shown that GLWamide neuropeptides, and for the first time sesquiterpenoid pathway genes could potentially be involved in the late phase of cnidarian tissue regeneration. The established sea anemone model will be useful for further investigation of biology and evolution in, and the effect of climate change on this important group of animals.

## Introduction

Regeneration, in general, refers to the replacement of lost or damaged structures and is widespread in diverse groups of animals (Bideau et al., 2021). Revealing the capacities and underlying mechanisms in different animals is important to understand both the evolution of regeneration, as well as their potential contributions to animal adaptation to the environment.

The phylum Cnidaria comprises of anthozoans (sea anemones, corals), hydrozoans (hydra), and medusozoans (jellyfishes), and plays important roles in both freshwater and marine environments (Slobodkin & Bossert, 2001; Enríquez et al., 2005; Pitt and Purcell, 2009; Litsios et al., 2012). In general, tissue regeneration in cnidarians is comparatively understudied than other animals such as planarians and vertebrates, and most of the detailed studies on the cellular and molecular mechanisms are on hydrozoans (Amiel et al., 2015). Diverse mechanisms were revealed between species and tissues. For instance, cell proliferation is not required for head and foot regeneration to occur in *Hydra* (Park et al., 1970), but a surge of stem cell proliferation occurs subsequent to decapitation and is crucial for head regeneration in *Hydractinia echinata* (Bradshaw et al., 2015). On the other hand, decapitation in *H. echinata* led to blastema proliferation in the area where the head used to be, but no blastema formed in stolon regeneration (Bradshaw et al., 2015). In other cnidarians, such as the sea anemone *Nematostella vectensis* (Layden et al., 2016; Zimmermann et al., 2020), coral *Favia favus* (Oren et al., 2001), jellyfish *Aurelia aurita* and *Clytia hemisphaerica* (Abrams et al., 2015; Sinigaglia et al., 2020), capabilities of regeneration of lost body parts have also been documented. Nevertheless, how non-coding RNAs contribute to cnidarian regeneration is poorly understood.

MicroRNAs (commonly called miRNAs) are small non-coding RNAs that are around 21–23 nucleotides long and are sequence-specific post-transcription regulators of gene expression in animals (Moran et al., 2017). In cnidarians, miRNAs predominantly have highly complementary messenger RNA targets and result in transcripts cleavage (Moran et al., 2014; Praher et al., 2021). Despite the fact that miRNAs have demonstrated their indispensable roles in regeneration in vertebrates such as mice and zebrafish, how they contribute to cnidarian tissue regeneration remains poorly explored. In limited studies such as that carried out in *H. magnipapillata*, small RNA transcriptomic analyses revealed 10 differentially expressed miRNAs during the process of head regeneration (Krishna et al., 2013).

**FIGURE 1:**
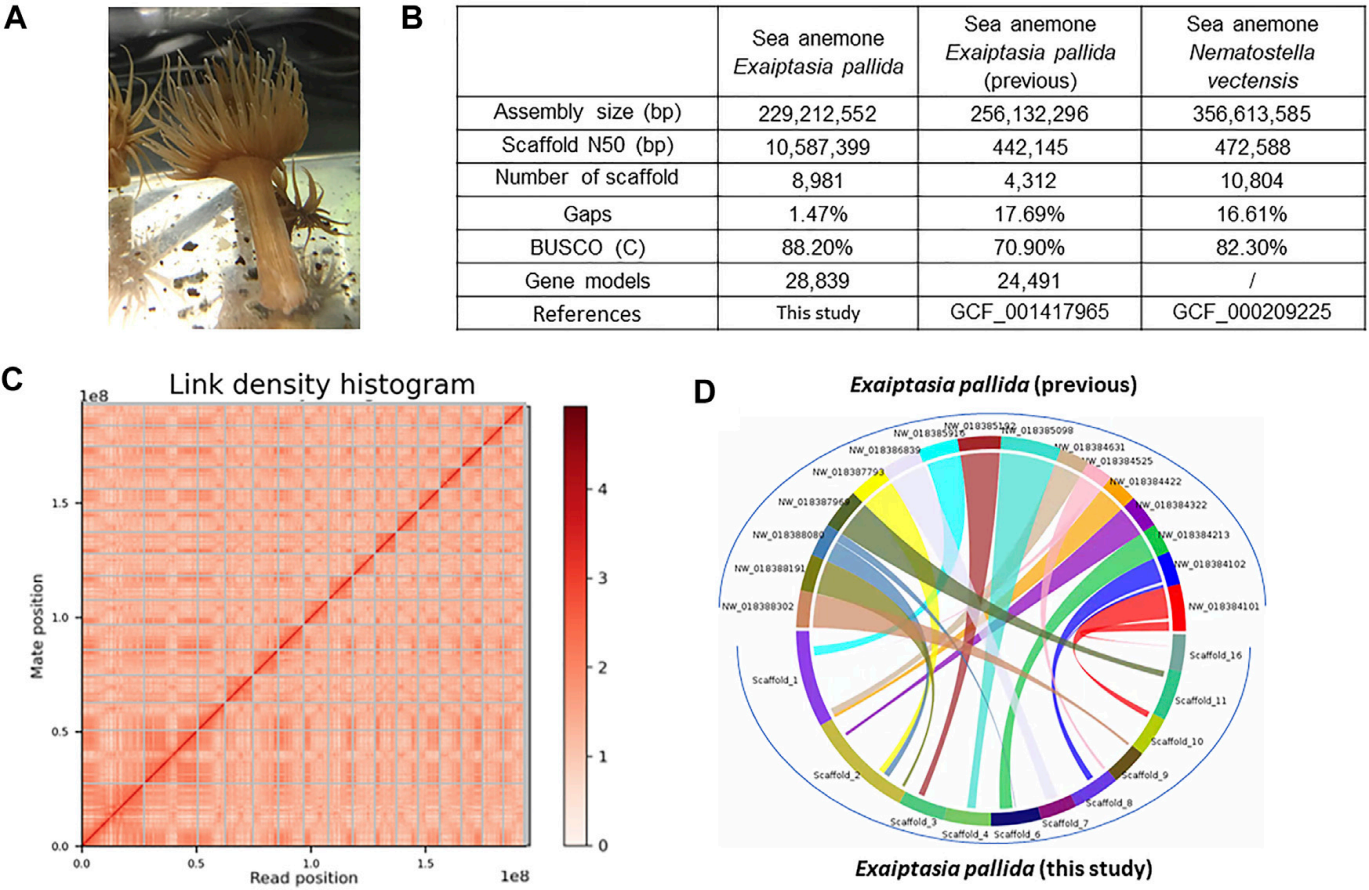
**(A)** *Exaiptasia pallida* individuals used in this study. **(B)** Genome sequencing information and statistics. **(C)** Hi-C information. The x- and y-axes give the mapping positions of the first and second read in the read pair respectively, grouped into bins. The color of each square gives the number of read pairs within that bin. Scaffolds less than 1 Mb are excluded; **(D)** Synteny blocks between the previous *Exapitasia diaphana* (GCF_001417965) genome and our genome.

In this study, a high-quality genome assembly of the sea anemone *Exaiptasia pallida* collected from Hong Kong waters (Figure 1A) is provided to form the basis of miRNA annotation in this cnidarian species. Both messenger RNA and miRNA transcriptomes were sequenced from 9 time points with triplicates during its tentacle regeneration to shed light on how these crucial genetic components contribute to regeneration in this important group of animals.

## Materials and methods

### Animal collection and husbandry

*E. pallida* were collected from Simon F.S. Li Marine Science Laboratory, The Chinese University of Hong Kong (CUHK) and kept in 30 ppt artificial sea water at 22°C. All of the individuals were collected from different colonies 1 day prior to experiments. To observe tentacle regeneration in *E. pallida*, 30 individuals had their tentacles cut at the base of the tentacles. The course of regeneration was observed and documented up to 23 days using a stereomicroscope.

### mRNA and small RNA transcriptomes

mRNA and small RNA extraction were done during 9 time points (0 h, 6 h, 12 h, 18 h, 1 day, 2, 3, 6, and 8 days) of *E. pallida* tentacle regeneration. Biological triplicates were done for each time point and each triplicate consisted of tissues excised from six individuals. Tissues from the base of tentacles were obtained for 0 h replicates and tissues from the tip of regenerating tentacles were obtained for the rest of the time points. mRNAs and small RNAs were extracted from the tissues obtained using TRIzol (Ambion) and miRVana Isolation kit respectively, and their concentrations were measured using NanoDrop™ One/One C Microvolume UV–Vis Spectrophotometer (Thermo Fisher Scientific, Fitchburg, WI). Agarose gel electrophoresis was used to assess the quality. Samples were stored at −80°C and sent to Novogene for transcriptome (Novaseq, PE151 platform for strand-specific library construction) and small RNA library (Novaseq, 50SE platform for strand-specific library construction) sequencing (Supplementary Tables S1,S4).

Transcriptome libraries were prepared using the NEBNext® Ultra™ Directional RNA Library Prep Kit for Illumina®. In brief, after the purification of mRNA with poly-T oligo-attached magnetic beads, the mRNA was fragmented randomly. Using random hexamer primer and M-MuLV Reverse Transcriptase (RNase H), the first cDNA strand was synthesized. The second cDNA strand was synthesized using DNA polymerase I and RNase H with the incorporation of dUTP. AMPure XP beads (Beckman Coulter, Beverly, United States) were used to purify the double-stranded cDNA to select cDNA fragments of 150–200 bp. Exonuclease/polymerase repaired the remaining overhangs of the cDNA into blunt ends. Subsequent to adenylation of 3′ ends of DNA fragments, NEBNext Adaptor was ligated to prepare for hybridization. The second strand of cDNA was digested by USER enzyme. The final library was generated by PCR amplification and purification of PCR products with AMPure XP beads.

Small RNA libraries were prepared using the NEBNext® Multiplex Small RNA Library Prep Set for Illumina®. In short, 3 and 5 adaptors were first ligated to the 3 and 5’ ends of small RNAs respectively. Subsequent to hybridization, the first strand of cDNA was synthesized with reverse transcription primers. Through PCR enrichment, the double-stranded library was generated. Purification was done to select fragments of 18–40 bp.

### Genome sequencing, Chicago and Omni-C libraries construction, and assembly

Genomic DNA was extracted from *E. pallida* using PureLink™ Genomic DNA Mini Kit (Invitrogen, Carlsbad, CA, United States). The oral disk was homogenized and digested for 2 h at 55°C. 10 times the amount suggested for PureLink™ Genomic Digestion Buffer, Proteinase K, RNase A, PureLink™ Genomic Lysis/Binding Buffer, and ethanol were used. The DNA was eluted with 35 μL ddH_2_O (concentration: 228.0 ng/μL; amount: 3.192 μg; OD260/280: 1.81; OD260/230: 1.91). The sample was then sent to Novogene for 10X Genomics linked-read sequencing and Dovetail Genomics for Chicago and Omni-C sequencing as described below. The details, including sequencing platform and prepared library size, are attached in Supplementary Table S2.

A Chicago library was made as described previously in Putnam et al. (2016). In short, high molecular genomic DNA was reconstructed into chromatin *in vitro* and fixed with formaldehyde solution. Resultant chromatin was then digested with the restriction enzyme DpnII. The 5’ overhangs were sealed with biotinylated nucleotides and the blunt ends were then ligated. The DNA was then purified from the crosslinked proteins and subject to the removal of biotins that were not located in the internal ligated fragments. Eventually, the DNA molecules were sheared to approximately 350 bp and a library was constructed using NEBNext Ultra enzymes and Illumina-compatible adaptors. Fragments that contain biotin were isolated by streptavidin beads prior to PCR enrichment of the library. The libraries were then sequenced on an Illumina HiSeq X platform to yield 161 million 2 × 150 bp paired end reads.

The Dovetail Omni-C library was prepared according to Manuals & Guides (https://dovetailgenomics.com/products/omni-c-product-page/). Briefly, the genomic DNA was fixed on site in the nucleus with formaldehyde solution prior to sample DNA extraction. Fixed chromatin was digested with DNAse I. The resultant chromatin ends were subject to end-repairing steps and ligated to biotinylated bridge adaptors and followed by proximity ligation. Afterwards, DNA was purified from the crosslinked proteins. The sequencing library was prepared the same way the Chicago library was. The sequencing platform produced 420 million 2 × 151 bp paired end reads in total, which provided 727x physical coverage of the genome (1–100 kbp).

The assembly was processed as described previously (Nong et al., 2020). Briefly, Kraken was used to check the 10X raw data for bacteria contamination (Wood & Salzberg, 2014). Using DSK (v 2.1.0), k-mers of the Illumina PE reads were counted with k = 21. In addition, based on a k-mer-based statistical approach in the GenomeScope webtool, estimation of genome size, heterozygosity, and repeat content were analyzed (Supplementary Table S2; GenomeScope.kmer_k21). Supernova (v 2.1.1; default parameters) generates phased and whole-genome *de novo* assemblies from a Chromium-prepared library (Weisenfeld et al., 2017). The *de novo* assembly and raw reads from the Chicago library were used as primary input data for HiRise (Putnam et al., 2016). Chicago library sequences were then aligned to the draft assembly using SNAP (http://snap.cs.berkeley.edu). The separation of Chicago read pairs mapped within draft scaffolds were analyzed by HiRise to generate a likelihood model for the estimation of genomic distance between read pairs, and the model was used to identify and break putative misjoints, score prospective joins, and make joins above a threshold. A similar procedure was adopted for OmniC data and eventually yielded a resultant genome.

### Transcriptome assembly and gene model annotation

The assembly and annotation were processed as described previously (Nong et al., 2020). Raw sequencing reads of the transcriptomes from different time points were quality-filtered by Trimmomatic (v0.33, with parameters “ILLUMINACLIP:TruSeq3-PE.fa:2:30:10 SLIDINGWINDOW:4:5 LEADING:5 TRAILING:5 MINLEN:25”; Bolger, Lohse, & Usadel, 2014). Using Funannotate, sequences of nuclear genomes were cleaned and masked. To align RNA-seq data by HISAT2 (v2.0.5; Kim et al., 2019), the soft-masked assembly was used to run “funannotate train” with the parameters “--stranded RF--max_intronlen 350000”. A genome-guided assembly was then generated with Trinity (v2.9.1). Transcript abundance was estimated using “--est_method RSEM—aln_method bowtie” (v1.1.2) with the script of “align_and_estimate_abundance.pl” of Trinity. TransDecoder (v5.0.2) was used to annotate coding regions within transcripts (Supplementary Tables S6,S7). Trinotate (v3.1.1) was used to perform functional annotation and analysis.

Contigs of the genome-guided assembly were also used to run PASA for prediction of gene models. In the “funannotate predict” step, GeneMark-ES v4.32 was used for ab initio gene prediction and Augustus was trained using BUSCO. The gene models from the prediction sources “Augustus”, high-quality Augustus predictions (HiQ), “pasa”, “snap”, “GlimmerHMM”, and “GeneMark” were passed to Evidence Modeler [EVM Weights: ('GeneMark’: 1, ‘HiQ’: 2, ‘pasa’: 6, ‘proteins’: 1, ‘Augustus’: 1, ‘GlimmerHMM’: 1, ‘snap’: 1, ‘transcripts’: 1)] to generate the final annotation files. Finally, EVidenceModeler was used to combine data from protein alignments, transcript alignments, and *ab initio* predictions to construct high quality evidence-based gene models. PASA was then used to update EVM consensus predictions and add UTR annotations and models for alternatively spliced isoforms. The protein-coding gene models were then searched in the NCBI nr database using BLASTp and in the swissprot database using diamond (v0.9.24) with parameters “--more-sensitive--evalue 1e-3”, and mapped by HISAT2 (version 2.1.0) with transcriptome reads (Supplementary Table S3). Gene matrix count tables were then generated by StringTie (version 2.1.1) and used for further differential gene expression analyses. The gene models with no homology to any known protein in the GenBank nr database and no mRNA support were removed from the final version. The differential gene expression analyses were performed by Trinity’s run_DE_analysis.pl and analyze_diff_expr.pl scripts using the edgeR method, where at least three replicates had CPM values ≥ 50, and cut-off values were set to 0.05 *p*-values (FDR) and 2-fold changes. Heatmaps were generated by PtR of Trinity, using the parameter “--min_rowSums 10 --log2 –CPM” and default complete gene and sample clustering methods.

### Gene family annotation

Putative gene sequences were retrieved from the established genome using the tBLASTn algorithm. The gene identity of each identified target was tested by comparing the sequences deposited in the NCBI nr database using the BLASTx algorithm. For neuropeptides, potential N-terminal signal peptide sequences were predicted by SignalP 3.0 (Bendtsen et al., 2004). The potential convertase cleavage sites were then manually checked and annotated based on the features of a canonical neuropeptide: various prohormone convertase cleavage (lysine/arginine) sites (KR, RR, R) in conjunction with a glycine residue (GKR, GRR, GR) for peptide C-terminal amidation. For phylogenetic analyses of different gene family sequences, amino acid sequences were aligned to other reference sequences extracted from NCBI; gapped sites were removed from alignments using the software MEGA 11 and phylogenetic trees (Neighbor-joining and Maximum likelihood) were constructed using MEGA 11 and IQ-TREE (http://www.iqtree.org/), with 1000 bootstrap replicates. The phylogenetic trees were further edited using iTOL (https://itol.embl.de/) and TBtools (Chen et al., 2020).

### Analysis of homeobox genes

**FIGURE 2:**
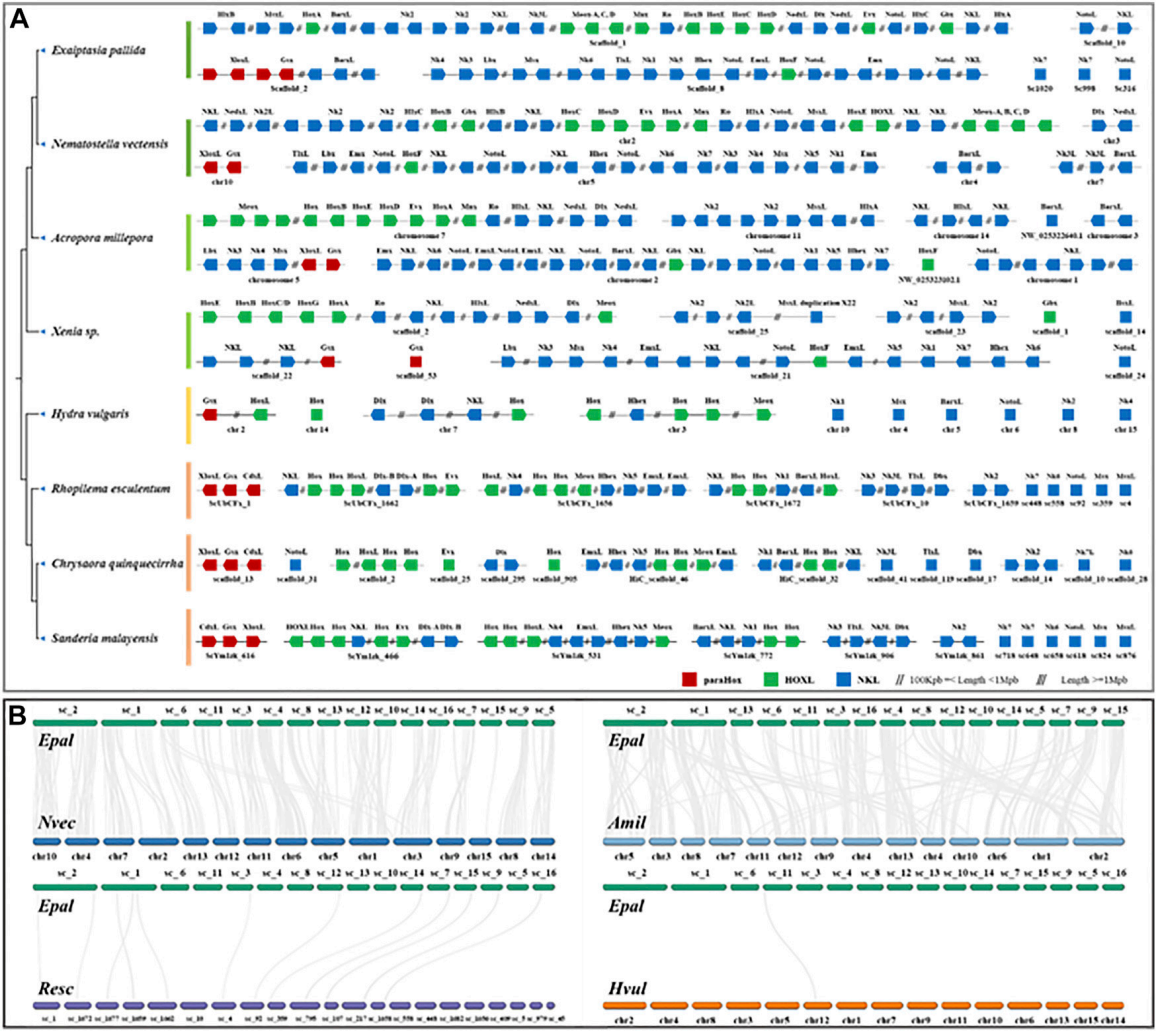
**(A)** Chromosomal organization of ANTP-class homeobox gene arrangement in cnidarian genomes. **(B)** Syntenic relationship between *Exaiptasia pallida*, *Nematostella vectensis* (sea anemone to sea anemone), *Acropora millepora* (sea anemone to coral), *Rhopilema esculentum* (sea anemone to jellyfish) and *Hydra vulgaris* (sea anemone to hydra).

Potential homeobox genes were identified by searching homeodomain sequences from *N. vectensis* (Ryan et al., 2006), *Branchiostoma floridae*, *Drosophila melanogaster*, and *Tribolium castaneum* (retrieved from HomeoDB; Zhong & Holland, 2011) in our *E. pallida* genome and transcriptome and previously published cnidarian genomes (Fuller et al., 2020; Hu et al., 2020; Nong et al., 2020; Zimmermann et al., 2020; Xia et al., 2021; Simakov et al., 2022) using tBLASTn (Supplementary Table S5). Then, NCBI CD-Search (Lu et al., 2020) was used to validate the presence of homeodomains in the retrieved sequences. Identification of each putative gene was tested by comparison to sequences confirmed in the NCBI nr database using BLASTx and BLASTp, phylogenetic analysis, and syntenic analysis (Figure 2B). Syntenic relationships between *E. pallida*, *N. vectensis*, *Acropora millepora*, *Rhopilema esculentum*, and *Hydra vulgaris* were computed using MCScanX with default parameters (Chen et al., 2020).

### Small RNA analysis

Small RNA-sequencing reads had their adaptor sequences trimmed and those with Phred quality score lower than 20 were removed. Using the mapper.pl script of miRDeep2, reads of length 18 to 27 bp were then mapped to respective genomes. miRDeep2 was used to identify novel miRNAs, which were checked manually whether they fulfill the criteria of MirGeneDB (http://mirgenedb.org/information). miRNAs which fulfill the criteria of MirGeneDB were then annotated to known miRNAs in the miRBase database according to sequence similarity. Expression profiling and quantification were performed by the quantifier.pl script of miRDeep2. MapMi was utilized to find potential miRNA loci in the genomes of *E. pallida* (MapMi scorer cutoff = 15). Matrix count tables were generated by miRDeep2’s script quantifier.pl with 0 mismatches in mapping reads to precursors and other default parameters. Different gene expression analyses were performed by Trinity’s run_DE_analysis.pl and analyze_diff_expr.pl scripts using the edgeR method, where at least three replicates had CPM values ≥ 50, and cut-offs were set to 0.05 *p*-values (FDR) and 2-fold changes.

### Synteny analyses

CIRCOS and synteny blocks were computed using SyMAP v5.0.6 (Synteny Mapping and Analysis Program) (Soderlund et al., 2011) with “mask_all_but_genes = 1’ to mask non-genic sequence” and other default parameters.

## Results

### High-quality *Exaiptasia pallida* genome

High molecular weight genomic DNA was extracted from one individual of *E. pallida* (Figure 1A) and sequenced on the Illumina short-read and 10X Genomics linked-read sequencing platforms. Chicago and Omni-C libraries were also established and sequenced on the Illumina platform (Supplementary Table S2). The genome sequences were primarily assembled with short raw reads. Subsequently, scaffolding was done using the Chicago and Omni-C reads. The genome assembly is 229.2 Mbp with a scaffold N50 of 10.6 Mbp (Figure 1B). This high physical contiguity is matched by high completeness, with 91.1% complete BUSCO score for metazoa_genes (version odb10) (Figure 1B). A total of 28,839 gene models including 454 tRNA genes and 28,385 protein coding genes, 95.78% and 98.48% of the encoded proteins, could hit the nr/swissprot databases and had mRNA support, respectively. The majority of the sequences assembled (∼84.18%) were contained in 16 pseudomolecules (Figure 1C). It is noticed that there is a chromosomal-level genome in the sea anemone *N. vectensis* available (Zimmermann et al., 2020), and our new resource represents the first near chromosomal-level genome generated for the species *E. pallida*.

### Homeobox clusters

Homeobox genes are essential players in body patterning and markers of large-scale genomic changes in evolution, and there is an increasing number of comparisons of homeobox genes between genomes in attempt to understand the evolution of these genes in animals (Chourrout et al., 2006; Holland et al., 2007; Holland, 2013; DuBuc et al., 2018; He et al., 2018). Analyses of homeobox gene organization across a range of species help to better understand the evolution of genome organization. The ANTP-class of homeobox genes is the largest group of homeobox genes in animals, including the ParaHox (*Gsx*, *Xlox*, and *Cdx*), Hox/Hox-linked (Hox genes, *Gbx*, *Mnx*, *Meox*, *Evx*, *En*, *Ro*, *Mnx*, *Hhex*, *Moex*, and *Dlx*), and NK/NK-linked clusters (*NK1*, *NK3*, *NK4*, *NK5*, *NK6*, *NK7*, *Msx*, *Lbx*, *Tlx*) (Holland, 2013; Ferrier, 2016). To investigate the evolution of gene clusters, we have annotated the ANTP-class of homeobox genes in a wide range of high-quality cnidarian genomes (Figure 2A, Supplementary Tables S2,S5).

In the genomes of bilaterians, the ANTP-class of homeobox genes are usually found in four chromosomal clusters (*ParaHox*, *Hox*, *NK*, and *NK2*) (Luke et al., 2003; Hui et al., 2012; Li et al., 2020). In a recent study, it was found that the Hox, NK, and Hox-like genes are clustered in two jellyfish genomes (Figure 2A; Nong et al., 2020), illustrating a divergent evolutionary pathway of the ANTP-gene cluster genes in cnidarians. Genomic analysis of the ANTP-class homeobox genes in *E. pallida* revealed that most of these genes are mainly located on three scaffolds similar to the jellyfish studies, including a ParaHox gene cluster (*Xlox* and *Gsx*) on Scaffold 2, Hox genes with *HlxL*, *MsxL*, *Meox*, *Ro*, *NedxL*, *Dlx, NK2* genes on Scaffold 1, and Hox gene with NK cluster (*NK1/3/4/5/6*, *Lbx*, *Msx*, *TlxL*, and *Hhex*) on Scaffold 8 (Figure 2A). The ANTP-class homeobox genes organization in this anthozoan further supported the previous findings in jellyfishes, and refuted that it is due to lineage-specific rearrangement. Nevertheless, the hypothetical mega-homeobox cluster organization as originally proposed by Pollard and Holland (2000) remains to be revealed in an extant animal.

### miRNA and messenger RNA profiles during tentacle regeneration

**FIGURE 3:**
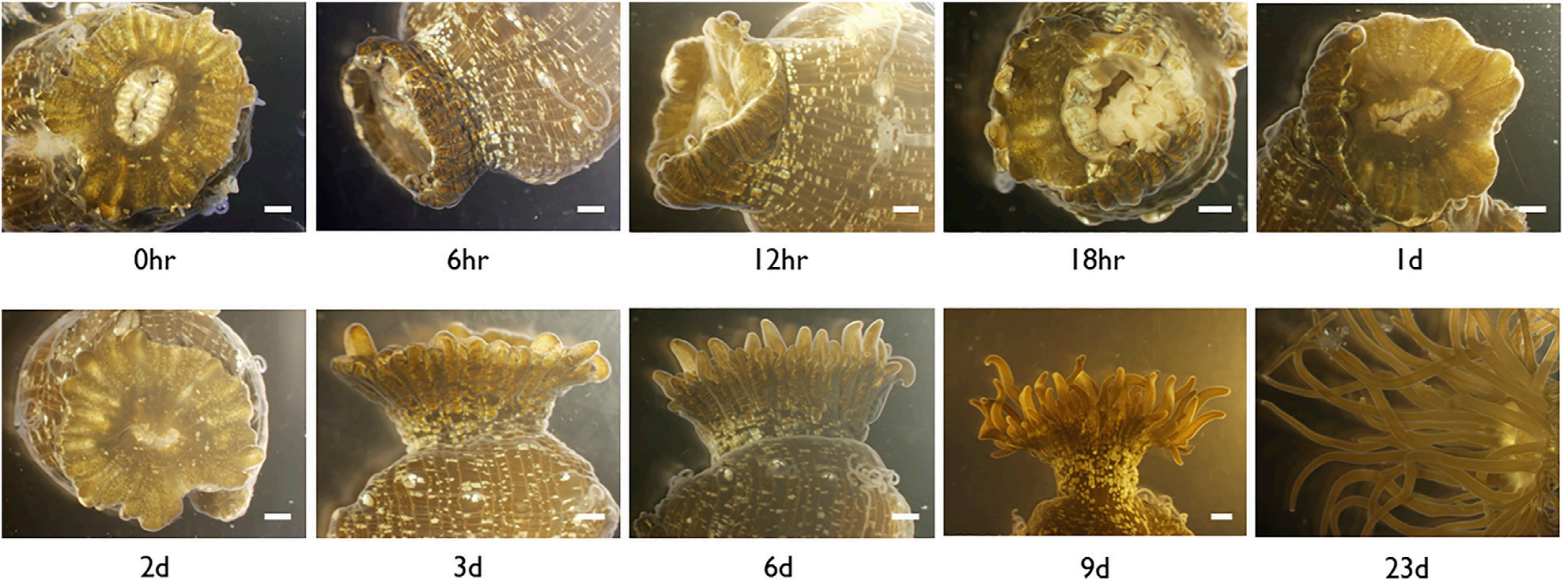
Microscopic images of *Exaiptasia pallida* cultured at 22°C with tentacles cut at the base from the moment of injury (0 h) to complete regeneration (Day 9). Scale bar, 1 mm.

**FIGURE 4:**
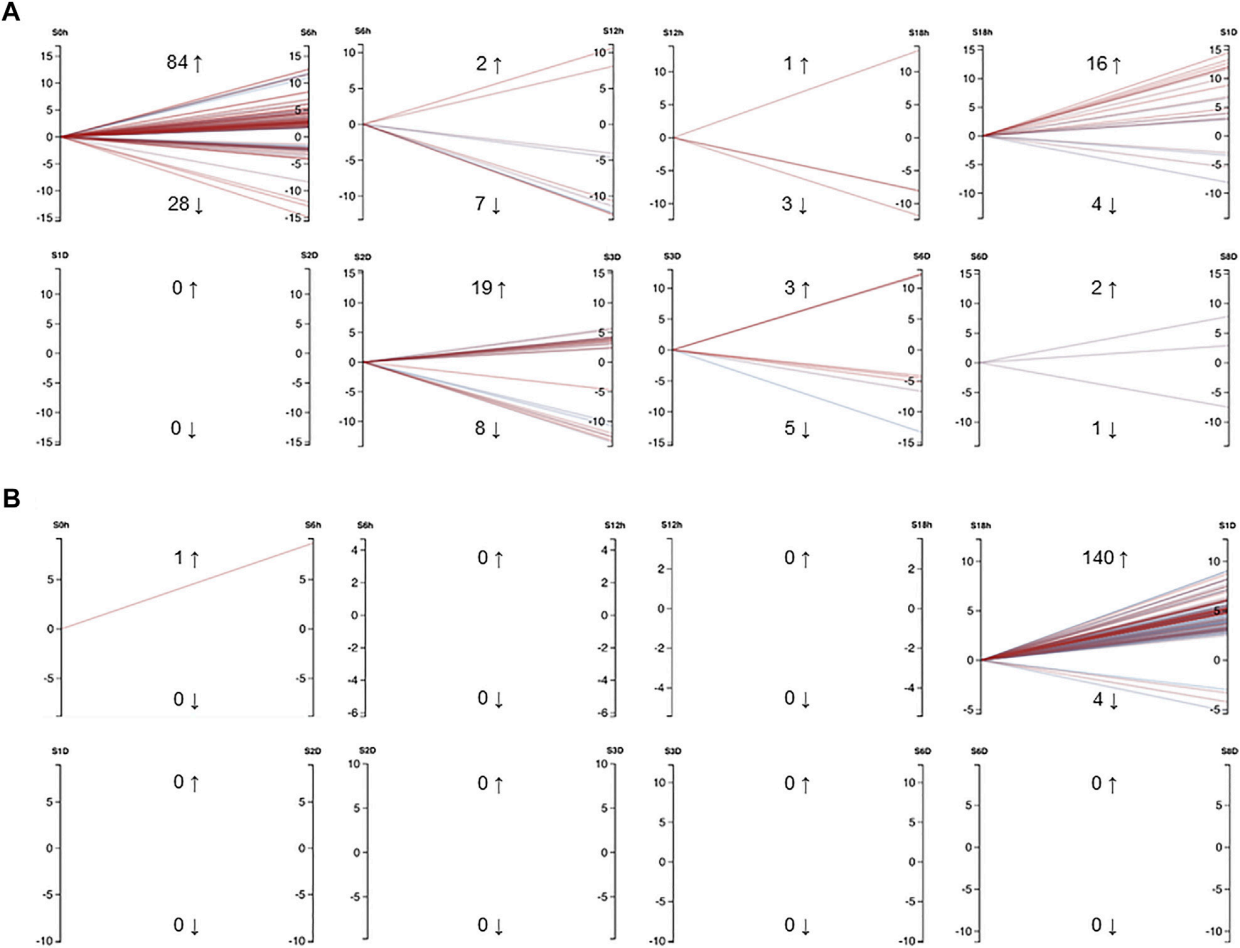
**(A)** Differentially expressed mRNAs in *Exaiptasia pallida* tentacle regeneration that had a minimum CPM of 50 in at least 3 samples. Cut-off values were set to FDR = 0.05, abs logFC = 1. **(B)** Differentially expressed miRNAs in *E. pallida* tentacle regeneration that had a minimum CPM of 5 in at least 3 samples. Cut-off values were set to FDR = 0.05, abs logFC = 1. Numbers adjacent to upwards and downwards arrows indicate the number of genes or miRNAs that were upregulated and downregulated respectively, e.g. from 0 hpa to 6 hpa, 84 mRNAs were upregulated and 28 mRNAs were downregulated. Each line is colored by FDR with red being more significant and blue less significant. S0h: 0 h post amputation (hpa), S6h: 6 hpa, S12h: 12 hpa, S18h: 18 hpa, S1D: 1 day post amputation (dpa), S2D: 2 dpa, S3D: 3 dpa, S6D: 6 dpa, S8D: 8 dpa. The graphs were generated using Degust (DOI: 10.5281/zenodo.3258932) (Powell, 2015).

In *E. pallida* individuals cultured at 22°C, the wound closed at 12 hpa (hours post-amputation) (Figure 3). At 18 hpa, tentacle buds became visible and grew longer at 2 and 3 dpa (days post-amputation) (Figure 3). A total of 54 small RNA and messenger RNA datasets representing 9 time points during *E. pallida* tentacle regeneration were generated (Supplementary Table S1). Across the 9 time points, 127 and 58 mRNAs were upregulated and downregulated respectively, and 141 and 4 miRNAs were upregulated and downregulated respectively (Figures 4A,B, Supplementary Figures S2–S4, Supplementary Tables S8–S10). Most of the differentially expressed mRNAs were differentially regulated between 0 hpa and 6 hpa, while the majority of the differentially expressed miRNAs were differentially regulated between 18 hpa and 24 hpa (Figures 4A,B).

We first investigated whether the key developmental and hormonal pathway genes were involved in *E. pallida* tentacle regeneration. Wnt signaling pathway genes are well known to be involved in the tissue regenerative processes in animals (e.g. Whyte et al., 2012). We found that the expression of *Frizzled*, *LRP*, *Wnt*, and other members in the Wnt signaling pathway were upregulated in the early- and mid-phase of tentacle regeneration (Supplementary Figures S5–S8, Supplementary Table S11).

**FIGURE 5:**
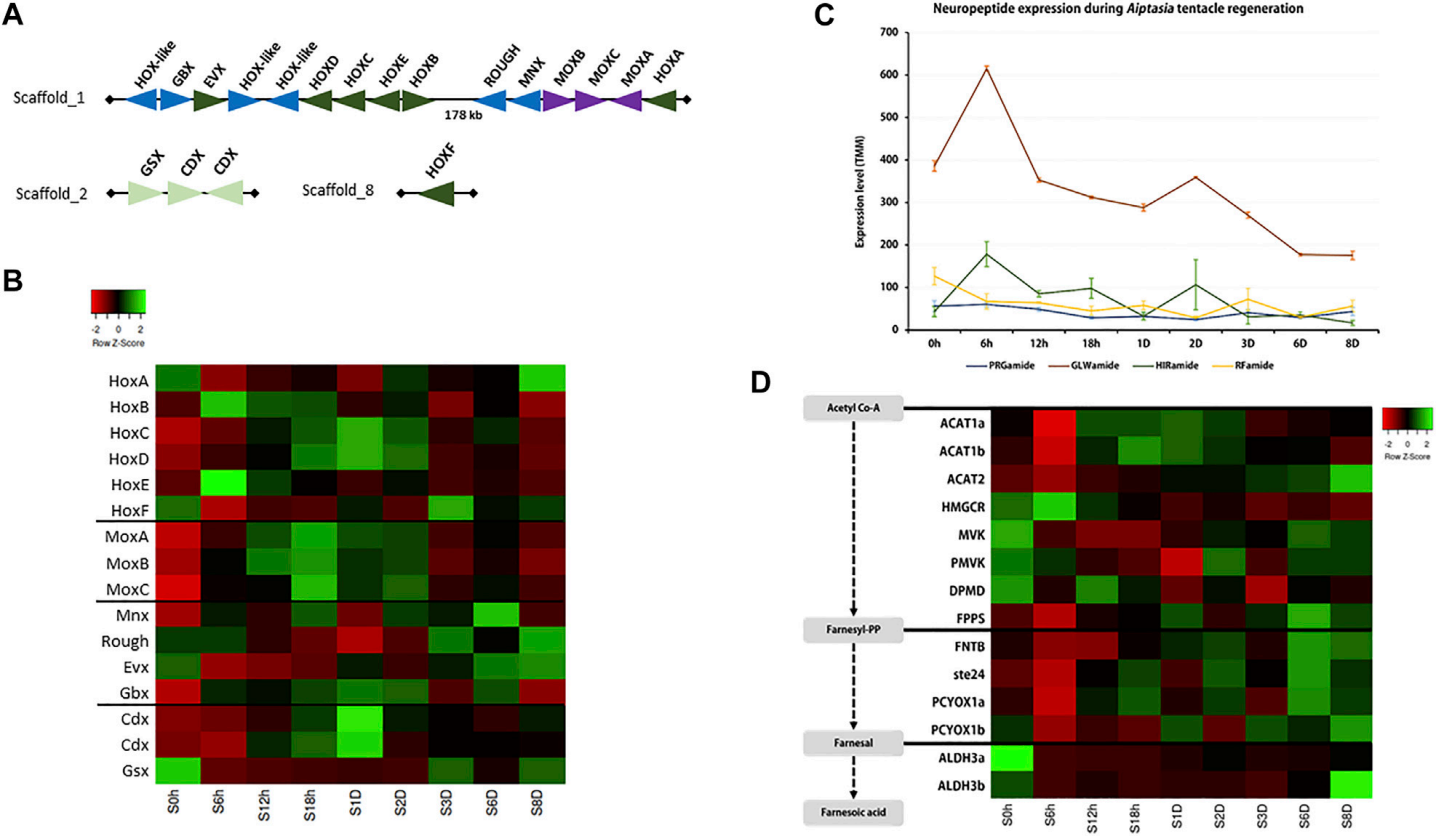
**(A)** Hox and other homeobox gene arrangement in the *Exaiptasia pallida* genome; **(B–D)** Expression of **(B)** Hox genes, **(C)** neuropeptides, and **(D)** sesquiterpenoid hormone pathway genes during tentacle regeneration.

Previous studies have identified the association of Hox gene expression in animal tissue regeneration (e.g. Novikova et al., 2016) so we investigated the expression of Hox/ParaHox genes during tentacle regeneration in *E. pallida* (Supplementary Figure S9). In *E. pallida*, a Hox cluster (*HoxA*-*HoxE*) together with *Mox*, *Gbx*, *Rough*, and *Msx* were identified on the same scaffold, while the ParaHox cluster, which consists of *Gsx* and 2 *Cdx*, was found on another scaffold (Figure 5A). During the regeneration of tentacles, a progressive upregulation of Hox genes was observed with *HoxB* and *HoxE* upregulated at 6 hpa, followed by the upregulation of *HoxC* and *HoxD* at 18 hpa (Figure 5B, Supplementary Table S12). *HoxF*, which is located on a different scaffold, was upregulated at 3 dpa (Figure 5B). Other homeobox genes including *Mox*, *Cdx*, and *Gbx* were upregulated between 18 hpa and 2 dpa, while *HoxA*, *Gsx*, *Mnx*, *Rough*, and *Evx* were downregulated shortly after tentacle amputation at 6 hpa (Figure 5B).

Neuropeptides play crucial roles as hormones and neurotransmitters and are involved in the regulation of a variety of developmental processes (e.g. Takahashi, 2020). Here, we annotated 4 cnidarian conserved neuropeptides: *PRGamide*, *GLWamide*, *HIRamide,* and *RFamide* (Figure 5C, Supplementary Figure S10, Supplementary Table S13). Among the four neuropeptides, *GLWamide* was found to be associated with the tentacle regenerative process in *E. pallida* and was upregulated at 6 hpa.

In a recent study, the sesquiterpenoid hormonal system was identified in cnidarians (Nong et al., 2020). To test whether sesquiterpenoids could potentially be involved in tissue regeneration in cnidarians, we investigated the expression of sesquiterpenoid biosynthetic pathway genes during tentacle regeneration in *E. pallida* (Supplementary Figures S11–S22, Supplementary Table S14). *Acetyl-CoA acetyltransferase* (*ACAT*) and the isoprenylation pathway genes (*FNTB*, *ste24*, *PCYOX1*) were downregulated, while upregulation of gene expression was observed for *HMGCR*, *MVK*, *PMVK*, *DPMD*, *FPPS*, *ALDH3a*, and *ALDH3b* at 6 hpa. At 6 dpa, both terpenoid backbone pathway and isoprenylation pathway genes were upregulated (Figure 5D).

## Discussion

Cnidarians such as the hydroid *Hydra* and the sea anemone *N. vectensis* are conventional animal models to understand tissue and body regeneration (Holstein et al., 2003; Havrilak et al., 2021; Röttinger, 2021). Hox genes were found to be regulated by miRNAs in the sea anemone *N. vectensis* (Moran et al., 2014). Prior to this study, there have been other attempts at utilizing the sea anemone *Exaiptasia* in understanding the regeneration processes (e.g. Singer, 1971; van der Burg et al., 2020; Presnell et al., 2022). Nevertheless, how miRNAs contribute to cnidarian regeneration is relatively understudied in cnidarians (Krishna et al., 2013). This study represents the first understanding of the underlying genetic regulation of miRNAs during tentacle regeneration in the sea anemone *E. pallida.*

To better annotate the miRNAs, especially the novel miRNAs, we have re-sequenced the *E. pallida* genome with a population collected naturally in Hong Kong and sequenced new small RNA transcriptomes. To date, there are a total of 103 assemblies of cnidarian genomes at NCBI (5 July 2022), and we have tested them with BUSCO v5.3 and the metazoa_odb10 database with the same default parameters (Supplementary Table S15). Forty of them had BUSCO scores between 90.2% and 96.1%. Synteny analysis between the previous *Exaiptasia diaphana* (interchangeable name for *E. pallida;* GCF_001417965; Baumgarten et al., 2015) genome and the newly assembled genome showed that 78.35% of the sequences assembled in GCA_001417965 are syntenous with the 16 pseudomolecules of our genome assembly (Figure 1D, Supplementary Table S15), representing a significantly improved genome assembly of this species. We also performed OrthoFinder on the longest genes in both genomes and found a total of 47,712 orthogroups, of which 3.14% was only found in GCA_001417965.1, 6.70% was only found in our new genome, and the remaining 90.16% was common (Supplementary Table S15). Our new genome will be useful for future understanding of cnidarian biology, in particular, genome biology and evolution.

miRNAs found in *Aiptasia* (Baumgarten et al., 2018), *N. vectensis* (Moran et al., 2014), *Sanderia malayensis* (Nong et al., 2020), *R. esculentum* (Nong et al., 2020), *Acropora digitifera* (Gajigan & Conaco, 2017), and *H. magnipapillata* (Krishna et al., 2013) were searched in the new *E. pallida* genome assembly. Ten miRNAs, namely miR-100, miR-2022, miR-2023, miR-2025, miR-2026, miR-2030, miR-2036, miR-2037, miR-2050, and miR-9425, are found amongst most anthozoans (Praher et al., 2021). Amongst the ten miRNAs, only one miRNA, miR-9425, was not identified in our *E. pallida* genome (Supplementary Table S16). All of the miRNAs found in *Aiptasia* (Baumgarten et al., 2018) can be found in the new genome assembly, and we observed a dynamic miRNA conservation between different cnidarians.

We have identified Wnt signaling pathway genes and homeobox genes that participate in tentacle regeneration, which are congruent with other cnidarian regeneration studies (e.g. Schummer et al., 1992; Guder et al., 20066; Stierwald et al., 2004; Trevino et al., 2011; Ramirez et al., 2022). *GLWamide*, one of the most studied neuropeptides, is responsive in tentacle regeneration of *E. pallida*. GLWamide was previously revealed to be involved in the process of muscle contraction in the sea anemone genus *Anthopleura* and the coral *Euphyllia ancora* (Takahashi & Hatta, 2011; Shikina et al., 2020), metamorphosis in *H. magnipapillata* (Takahashi & Hatta, 2011), settlement in the coral *Acropora palmata* (Erwin & Szmant 2010; Takahashi & Hatta, 2011), and transition timing modulation of *N. vectensis* from planula larvae to polyps (Nakanishi & Martindale, 2018). Given its participation in cnidarian tissue regeneration, this study has now revealed that the level of pleiotropy of GLWamide is higher than previously thought. Another interesting finding of this study is that we have explored the potential roles of the newly identified sesquiterpenoid hormones in cnidarians (Nong et al., 2020). Notably, genes in both the terpenoid backbone pathway and isoprenylation pathway were upregulated in the latter phase of regeneration. This is the first study showing the potential roles of sesquiterpenoids in cnidarian regeneration.

Common DEGs among tentacle regeneration of *E. pallida* from our study and body regeneration of *E. pallida* from the study conducted by van der Burg et al. (2020) were identified and their expression levels at different time points were analysed. The study conducted by van der Burg et al. (2020) and this study investigated different regeneration time points, thus the most similar time points were compared. The expression levels of 9 common DEGs (genes with FDR smaller than or equal to 0.05 and abs logFC greater than or equal to 1) at 0 hpa (from both studies), 6 hpa (this study) VS. 8 hpa (van der Burg et al., 2020), 18 hpa (this study) VS. 20 hpa (van der Burg et al., 2020), 48 hpa (from both studies), and 72 hpa (from both studies) were compared. All 9 of these common genes were differentially expressed from 0 hpa to 6 hpa, or from 0 hpa to 8 hpa. No common DEGs were found in other time points. C3 and PZP-like alpha-2-macroglobulin domain-containing protein 8 (CPAMD8), protein naked cuticle homolog 2-like (nkd2l), and patched domain-containing protein 3 (PTCHD3) displayed significant upregulation from 0 hpa to 6/8 hpa (Supplementary Figures S23–S25). *E. pallida* displayed a similar trend in the expression of CPAMD8 and nkd2l across different time points in our study and the study conducted by van der Burg et al. (2020). For most of the transcripts, both genes displayed an upregulation from 0 hpa to 6 hpa, followed by gradual downregulation in subsequent time points. However, the read counts reported by van der Burg et al. (2020) are significantly lower than that by our study. The species displayed a somewhat similar trend in the expression of PTCHD3 across different time points in our study and the study conducted by van der Burg et al. (2020). Gene1884 and gene8054 were both annotated as PTCHD3 van der Burg et al. (2020). Gene1884 was upregulated from 0 hpa to 8 hpa and was gradually downregulated in subsequent time points van der Burg et al. (2020), which is the same trend as that observed in our study. On the other hand, gene8054 was downregulated from 0 hpa to 8 hpa and was upregulated until a downregulation at 72 hpa. Advillin, heme-binding protein 2 (HEBP2), and protein NLRC5 were significantly downregulated from 0 hpa to 6/8 hpa (Supplementary Figures S26–S28). *E. pallida* displayed a similar trend in the expression of advillin across time points in our study and the study conducted by van der Burg et al. (2020), with an exception observed from 18/20 hpa to 48 hpa. The general trend in the expression of HEBP2 observed in this study is also the same as that in the study conducted by van der Burg et al. (2020). A similar trend in the expression of protein NLRC5 across time points was observed, with an exception observed from 6/8 hpa to 18/20 hpa. TNF receptor-associated factor 3 (TRAF3) expression displayed opposite trends from 0 hpa to 6/8 hpa, with significant upregulation and downregulation (Supplementary Figure S29). Fibroblast growth factor receptor (FGFR) expression displayed opposite trends from 0 hpa to 6/8 hpa, with significant downregulation and upregulation (Supplementary Figure S30). La-related protein 6 (LARP6) displayed significant changes in expression in different time points in our study and the study conducted by van der Burg et al. (2020) (Supplementary Figure S31). The overall trend of expression is similar with an exception observed from 48 hpa to 72 hpa. None of the DEGs identified in oral and physal regeneration in *N. vectensis* (dissected halfway between the oral and aboral ends; Schaffer et al., 2016) were found in tentacle regeneration of *E. pallida* (this study) and whole body regeneration of *E. pallida* (dissected perpendicular to the oral-aboral axis; van der Burg et al. 2020).

CPAMD8 has a Kazal-type serine proteinase inhibitor/follistatin-like domain (Li et al., 2004). This gene has been found to be upregulated in regeneration-incompetent iris in newts and axolotls (Sousounis et al., 2013; Sousounis et al., 2014). Interestingly, CPAMD8 was significantly upregulated from 0 hpa to 6/8 hpa in both our study and the study done by van der Burg et al. (2020). Naked cuticle (Nkd) is a gene that targets Dishevelled to antagonize Wingless, a signal for the Wnt signaling pathway (Zeng et al., 2000; Rousset et al., 2001). It may also activate a second Wnt signaling pathway that controls planar cell polarity (Creyghton et al., 2005). Nkd2l was significantly upregulated at 6 hpa in our study and 8 hpa in the study conducted by van der Burg et al. (2020), suggesting that during these time points, nkd2l targeted Dishevelled to antagonize Wingless and the Wnt signaling pathway that controls planar cell polarity was activated. Patched is a receptor that mediates the Hedgehog (Hh) signaling pathway, which is involved in blastema cell proliferation, dorsoventral patterning, cartilage induction, and regulation of progenitor and stromal cell fate during regeneration (Schnapp et al., 2005; Ochoa et al., 2010). Patched has been shown to be crucial for posterior specification during planarian body regeneration (Yazawa et al., 2009). PTCHD3 was significantly upregulated at 6 hpa in our study and 8 hpa in the study conducted by van der Burg et al. (2020), suggesting that Hh signaling occurred during these time points. Advillin is a member of the gelsolin/villin family of actin regulatory proteins (Marks et al., 1998), and is involved in actin bundling (Rao et al., 2017). Actin cytoskeleton has been shown to be required for regeneration in previous studies (e.g. Lahne et al., 2015). Advillin is expressed at the tips of filopodia during axon regeneration in mice (Chuang et al., 2018). In our study and the study conducted by van der Burg et al., (2020), the expression level of the gene was significantly reduced post-amputation. HEBP2 facilitates necrotic cell death (Szigeti et al., 2006). This gene was significantly downregulated at 6 hpa in our study and 8 hpa in the study conducted by van der Burg et al. (2020), suggesting that necrosis was suppressed during these time points. Protein NLRC5 negatively regulates the NF-kappa-B and type I interferon signaling pathways in macrophages and decreases innate cytokine production and antiviral immunity (Cui et al., 2010; Tong et al., 2012). Immune genes have been found to be differentially expressed during regeneration in previous studies (e.g. Stewart et al., 2017). Protein NLRC5 was significantly downregulated at 6 hpa in our study and 8 hpa in the study conducted by van der Burg et al. (2020), suggesting that the immune system also has a vital role in regeneration in *E. pallida*. Degradative ubiquitination of TRAF3 during MyD88-depedent TLR signaling is crucial for the activation of mitogen-activated protein kinases (MAPKs) (Tseng et al., 2010), which have been found to play a role in regeneration in many regeneration studies (e.g. Jopling et al., 2012). TRAF3 also activates NF-kappa-B (Gardam et al., 2008) and regulates B cell survival (Gardam et al., 2008) and the production of type I interferons and proinflammatory cytokines (Tseng et al., 2010), demonstrating its importance in immunoresponses. Intriguingly, TRAF3 showed different trends of expression from 0 hpa to 6/8 hpa during tentacle regeneration (our study) and whole body regeneration (van der Burg et al. 2020) and its expression continued to increase until 18 hpa in our study, suggesting that MAPKs and immunoresponses may have a more important role in body regeneration and tentacle regeneration in *E. pallida* respectively. *Fgfr2* and *fgfr4* expression were detected in epicardial tissue during zebrafish heart regeneration (Lepilina et al., 2006). While wound fibrin was displaced with myocardium by 30 dpa in wild-type ventricles, dominant-negative Fgfr individuals retained considerable amounts of fibrin and developed scars that were large and rich in collagen, demonstrating that zebrafish heart regeneration is Fgf-dependent (Lepilina et al., 2006). FGFR was significantly downregulated and upregulated at 6 hpa in our study and at 8 hpa in the study conducted by van der Burg et al. (2020) respectively, suggesting that Fgfr signaling may be involved in whole body regeneration but not tentacle regeneration in *E. pallida*. LARP6 was found to be statistically upregulated during whole body regeneration in the sea anemone *Calliactis polypus* (Stewart et al., 2017). The gene is important for the interaction between collagen mRNAs and vimentin filaments and the stabilization of collagen mRNAs (Challa & Stefanovic, 2011). LARP6 was significantly upregulated at 6 hpa in our study and 8 hpa in the study conducted by van der Burg et al. (2020), suggesting that collagen mRNAs and vimentin filaments may also have important roles in regeneration in *E. pallida*.

An intriguing phenomenon we have observed in the 54 mRNA and miRNA transcriptomes is that the majority of the mRNAs were differentially expressed in the earliest phase of tentacle regeneration, while there was a delay of timing before most of the miRNAs were differentially regulated. These data imply that early tissue regeneration in *E. pallida* is controlled by mRNAs, and miRNAs mainly serve in the second phase of tissue regeneration by interacting with the mRNAs. Whether the roles of miRNAs in this second phase is “fine-tuning” the expression of mRNAs in the first phase, or act as a transitional phase to induce genes in another phase, or performing both functions, remains to be carefully examined. It is also unclear whether the interesting phenomenon in which mRNAs are differentially expressed in the earliest phase of regeneration and the differential regulation of miRNAs is delayed to a later phase is *E. pallida* specific or applicable to other cnidarians, which warrants further investigation.

This study has established the sea anemone *E. pallida* as a cnidarian model with a high-quality genome to study the contribution of miRNAs in tentacle regeneration. Sea anemones are well known to be sensitive to temperature changes and are more prone to bleach under increased temperatures. In *Hydra* and *N. vectensis,* temperature could affect their regeneration processes (Miljkovic-Licina et al., 2007; Kü et al., 2008; Amiel et al., 2015). Given the relatively fast tentacle regeneration time in *E. pallida*, alongside other conventional cnidarian models, we propose that this species can also be further used to test how physical parameters such as temperature, oxygen, nitrogen, and carbon dioxide content affect tissue regeneration and understand the effects of climate change on cnidarians.

## Data Availability

The datasets presented in this study can be found in online repositories. The names of the repository/repositories and accession number(s) can be found below: Bioproject accession number: PRJNA631085.
