# Supplementary material for: Genome of the sea anemone Exaiptasia pallida and transcriptome profiles during tentacle regeneration
Source: Front Cell Dev Biol. 2022 Aug 17;10:900321. doi: 10.3389/fcell.2022.900321 (PMC9444052; doi:10.3389/fcell.2022.900321)
Supplement: Supplementary file 1 [file DataSheet3.docx]

Supplementary Table 4 – sRNA length distribution

Supplementary Table 5 – Annotation of homeobox genes in various cnidarians

Supplementary Table 6 – mRNA annotation

Supplementary Table 7 – mRNA sequences

Supplementary Table 8 – Differential gene expression of mRNAs during *Exaiptasia pallida*

regeneration

Supplementary Table 9 – Annotated microRNAs in *Exaiptasia pallida* and their gene expression

during regeneration

Supplementary Table 10 – Expression of genes and microRNAs mentioned in Supplementary Figure 4

during *Exaiptasia pallida* regeneration

Supplementary Table 11 – Expression of Wnt signaling pathway genes mentioned in Supplementary

Figure 5 during *Exaiptasia pallida* regeneration

Supplementary Table 12 – Hox gene annotation and their gene expression during *Exaiptasia pallida*

regeneration

Supplementary Table 13 – Neuropeptide annotation and their gene expression during *Exaiptasia*

*pallida* regeneration

Supplementary Table 14 – Sesquiterpenoid biosynthetic pathway gene annotation and their

expression during *Exaiptasia pallida* regeneration

Supplementary Table 15 – BUSCO scores of cnidarian genomes at NCBI and *Exaiptasia pallida*

genome synteny and orthogroups

Supplementary Table 16 – Presence of microRNAs in various cnidarians
