# Supplementary material for: Genome of the sea anemone Exaiptasia pallida and transcriptome profiles during tentacle regeneration
Source: Front Cell Dev Biol. 2022 Aug 17;10:900321. doi: 10.3389/fcell.2022.900321 (PMC9444052; doi:10.3389/fcell.2022.900321)
Supplement: Supplementary file 3 [file DataSheet1.docx]

**Supplementary Tables and Figures**

Supplementary Table 1. *Exaiptasia pallida* transcriptome sequencing data information

Supplementary Table 2. *Exaiptasia pallida* genome sequencing data information

Supplementary Table 3. Mapping rates of transcriptome libraries

Supplementary Figure 1. Phylogenetic tree of ANTP-class homeobox genes

Supplementary Figure 2. Heatmap showing the expression of genes during different time points of *Exaiptasia pallida* tentacle regeneration

Supplementary Figure 3. Heatmap showing the expression of miRNAs during different time points of *Exaiptasia pallida* tentacle regeneration

Supplementary Figure 4. Lists of genes and microRNAs that show differential expression during different time points of *Exaiptasia pallida* tentacle regeneration

Supplementary Figure 5. Heatmaps showing the expression of Wnt signaling pathway genes during different time points of *Exaiptasia pallida* tentacle regeneration

Supplementary Figure 6. Phylogenetic tree of Frizzled genes in *Exaiptasia pallida*

Supplementary Figure 7. Phylogenetic tree of LRP genes in *Exaiptasia pallida*

Supplementary Figure 8. Phylogenetic tree of Wnt genes in *Exaiptasia pallida*

Supplementary Figure 9. Phylogenetic tree of Hox/ParaHox genes in *Exaiptasia pallida*

Supplementary Figure 10. Neuropeptide sequences identified in *Exaiptasia pallida*

Supplementary Figure 11. Phylogenetic tree of *acetyl-CoA C-acetyltransferase* (*ACAT*)

Supplementary Figure 12. Phylogenetic tree of *hydroxymethylglutaryl-CoA synthase* (*HMGCS*)

Supplementary Figure 13. Phylogenetic tree of *hydroxymethylglutaryl-CoA reductase* (*HMGCR*)

Supplementary Figure 14. Phylogenetic tree of *mevalonate kinase* (*MK*) and *phosphomevalonate kinase* (*PMK*)

Supplementary Figure 15. Phylogenetic tree of *diphosphomevalonate decarboxylase* (*DPMD*)

Supplementary Figure 16. Phylogenetic tree of *isopentenyl-diphosphate delta-isomerase* (*IPPI*)

Supplementary Figure 17. Phylogenetic tree of *farnesyl pyrophosphate synthase* (*FPPS*)

Supplementary Figure 18. Phylogenetic tree of *farnesyltransferase beta* (*FNTB*)

Supplementary Figure 19. Phylogenetic tree of *ste24 endopeptidase* (*ste24*)

Supplementary Figure 20. Phylogenetic tree of *protein-S-isoprenylcysteine O methyltransferase* (*ste14*/*ICMT*)

Supplementary Figure 21. Phylogenetic tree of *prenylcysteine oxidase* (*PCYOX1*)

Supplementary Figure 22. Phylogenetic tree of *aldehyde dehydrogenase 3* (*ALDH3*)

Supplementary Figure 23. Temporal expression of *C3 and PZP-like alpha-2-macroglobulin*

*domain-containing protein 8* (*CPAMD8*)

Supplementary Figure 24. Temporal expression of *protein naked cuticle homolog 2-like*

(*nkd2l*)

Supplementary Figure 25. Temporal expression of *patched domain-containing protein 3*

(*PTCHD3*)

Supplementary Figure 26. Temporal expression of *advillin*

Supplementary Figure 27. Temporal expression of *heme-binding protein 2* (*HEBP2*)

Supplementary Figure 28. Temporal expression of protein *NLRC5*

Supplementary Figure 29. Temporal expression of *TNF receptor-associated factor 3* (*TRAF3*)

Supplementary Figure 30. Temporal expression of *fibroblast growth factor receptor*(*FGFR*)

Supplementary Figure 31. Temporal expression of *la-related protein 6* (*LARP6*)

**Supplementary Table 1. *Exaiptasia pallida* transcriptome sequencing data information.**

| **Type** | **Platform** | **Samples** | **replica** | **No.CleanReads** | **No.CleanBases** | **Accession number** |
| --- | --- | --- | --- | --- | --- | --- |
| mRNA | Novaseq PE150 | S0h | S0h_12 | 40,830,094 | 6,124,387,913 | SRR13854868 |
|  |  |  | S0h_13 | 39,938,800 | 5,990,693,278 | SRR13854867 |
|  |  |  | S0h_17 | 45,986,844 | 6,898,018,534 | SRR13854856 |
|  |  | S12h | S12h_1 | 47,464,526 | 7,119,652,099 | SRR13854848 |
|  |  |  | S12h_2 | 44,759,952 | 6,713,986,564 | SRR13854847 |
|  |  |  | S12h_3 | 44,459,732 | 6,668,955,455 | SRR13854846 |
|  |  | S18h | S18h_1 | 38,226,590 | 5,733,984,918 | SRR13854845 |
|  |  |  | S18h_2 | 39,266,968 | 5,890,039,531 | SRR13854844 |
|  |  |  | S18h_3 | 44,750,402 | 6,712,554,228 | SRR13854843 |
|  |  | S1D | S1D_2 | 38,901,546 | 3,889,446,791 | SRR13854842 |
|  |  |  | S1D_3 | 38,290,314 | 3,795,982,885 | SRR13854866 |
|  |  |  | S1D_6 | 43,555,690 | 6,533,349,121 | SRR13854865 |
|  |  | S2D | S2D_1 | 71,710,658 | 10,756,580,760 | SRR13854864 |
|  |  |  | S2D_5 | 40,709,228 | 6,106,378,864 | SRR13854863 |
|  |  |  | S2D_6 | 40,351,418 | 6,052,586,500 | SRR13854862 |
|  |  | S3D | S3D_1 | 45,100,064 | 4,509,191,292 | SRR13854861 |
|  |  |  | S3D_3 | 45,290,330 | 4,497,584,550 | SRR13854860 |
|  |  |  | S3D_4 | 42,180,970 | 6,327,139,654 | SRR13854859 |
|  |  | S6D | S6D_4 | 57,343,716 | 8,601,543,213 | SRR13854858 |
|  |  |  | S6D_5 | 43,639,248 | 6,545,878,949 | SRR13854857 |
|  |  |  | S6D_6 | 42,577,884 | 6,386,681,684 | SRR13854855 |
|  |  | S6h | S6h_1 | 44,958,180 | 6,743,644,980 | SRR13854854 |
|  |  |  | S6h_2 | 54,776,198 | 8,216,330,152 | SRR13854853 |
|  |  |  | S6h_3 | 38,763,874 | 5,814,326,869 | SRR13854852 |
|  |  | S8D | S8D_1 | 49,111,996 | 4,869,401,677 | SRR13854851 |
|  |  |  | S8D_3 | 52,690,740 | 7,903,444,152 | SRR13854850 |
|  |  |  | S8D_4 | 42,908,802 | 6,436,220,836 | SRR13854849 |
| small RNA | Novaseq SE50 | S0h | S0h_12 | 25,185,385 | 1,259,269,250 | SRR13855323 |
|  |  |  | S0h_13 | 24,632,250 | 1,231,612,500 | SRR13855322 |
|  |  |  | S0h_17 | 20,102,991 | 1,005,149,550 | SRR13855311 |
|  |  | S12h | S12h_1 | 25,709,636 | 1,285,481,800 | SRR13855303 |
|  |  |  | S12h_2 | 25,846,042 | 1,292,302,100 | SRR13855302 |
|  |  |  | S12h_3 | 28,492,054 | 1,424,602,700 | SRR13855301 |
|  |  | S18h | S18h_1 | 30,608,851 | 1,530,442,550 | SRR13855300 |
|  |  |  | S18h_2 | 31,862,157 | 1,593,107,850 | SRR13855299 |
|  |  |  | S18h_3 | 24,567,210 | 1,228,360,500 | SRR13855298 |
|  |  | S1D | S1D_2 | 28,145,403 | 743,245,260 | SRR13855297 |
|  |  |  | S1D_3 | 46,618,099 | 1,170,691,431 | SRR13855321 |
|  |  |  | S1D_6 | 24,284,116 | 1,214,205,800 | SRR13855320 |
|  |  | S2D | S2D_1 | 24,570,491 | 687,565,115 | SRR13855319 |
|  |  |  | S2D_5 | 20,679,617 | 1,033,980,850 | SRR13855318 |
|  |  |  | S2D_6 | 22,783,780 | 1,139,189,000 | SRR13855317 |
|  |  | S3D | S3D_1 | 35,834,920 | 854,805,994 | SRR13855316 |
|  |  |  | S3D_3 | 22,554,201 | 601,348,986 | SRR13855315 |
|  |  |  | S3D_4 | 22,210,078 | 1,110,503,900 | SRR13855314 |
|  |  | S6D | S6D_4 | 24,477,994 | 671,768,163 | SRR13855313 |
|  |  |  | S6D_5 | 24,059,045 | 1,202,952,250 | SRR13855312 |
|  |  |  | S6D_6 | 20,661,474 | 1,033,073,700 | SRR13855310 |
|  |  | S6h | S6h_1 | 36,909,552 | 1,845,477,600 | SRR13855309 |
|  |  |  | S6h_2 | 27,595,317 | 1,379,765,850 | SRR13855308 |
|  |  |  | S6h_3 | 22,313,626 | 1,115,681,300 | SRR13855307 |
|  |  | S8D | S8D_1 | 48,671,493 | 1,324,505,909 | SRR13855306 |
|  |  |  | S8D_3 | 23,955,321 | 1,197,766,050 | SRR13855305 |
|  |  |  | S8D_4 | 23,309,696 | 1,165,484,800 | SRR13855304 |

**Supplementary Table 2. *Exaiptasia pallida* genome sequencing data information.** **Assembled genome size = 229,212,552 bp.

| **Platform** | **Read length(bp)** | **Library size** | **Reads** | **Bases** | **Coverage**** |
| --- | --- | --- | --- | --- | --- |
| HiSeq4000 150PE | 150 | 300bp | 163250516 | 24,487,577,400 | 107 |
| HiseqXten 150PE (Chromium WGS) | 150 | 50kp | 189,298,992 | 28,394,848,800 | 124 |
| CHiCAGO | 150 | 1-100kb | 321,633,672 | 48,245,050,800 | 210 |
| Omni-C | 150 | 100-1000kb | 240,964,692 | 36,144,703,800 | 158 |

**Supplementary Table 3. Mapping rates of transcriptome libraries.**

| **Sample** | **Replica** | **Overall alignment rate** |
| --- | --- | --- |
| S0h | S0h_12 | 79.91% |
|  | S0h_13 | 77.50% |
|  | S0h_17 | 78.53% |
| S12h | S12h_1 | 80.24% |
|  | S12h_2 | 81.90% |
|  | S12h_3 | 80.99% |
| S18h | S18h_1 | 83.19% |
|  | S18h_2 | 81.83% |
|  | S18h_3 | 81.47% |
| S1D | S1D_2 | 89.72% |
|  | S1D_3 | 77.89% |
|  | S1D_6 | 80.52% |
| S2D | S2D_1 | 80.96% |
|  | S2D_5 | 85.29% |
|  | S2D_6 | 82.85% |
| S3D | S3D_1 | 88.00% |
|  | S3D_3 | 83.34% |
|  | S3D_4 | 84.46% |
| S6D | S6D_4 | 82.49% |
|  | S6D_5 | 86.44% |
|  | S6D_6 | 87.04% |
| S6h | S6h_1 | 79.23% |
|  | S6h_2 | 80.13% |
|  | S6h_3 | 79.99% |
| S8D | S8D_1 | 85.28% |
|  | S8D_3 | 78.93% |
|  | S8D_4 | 83.09% |


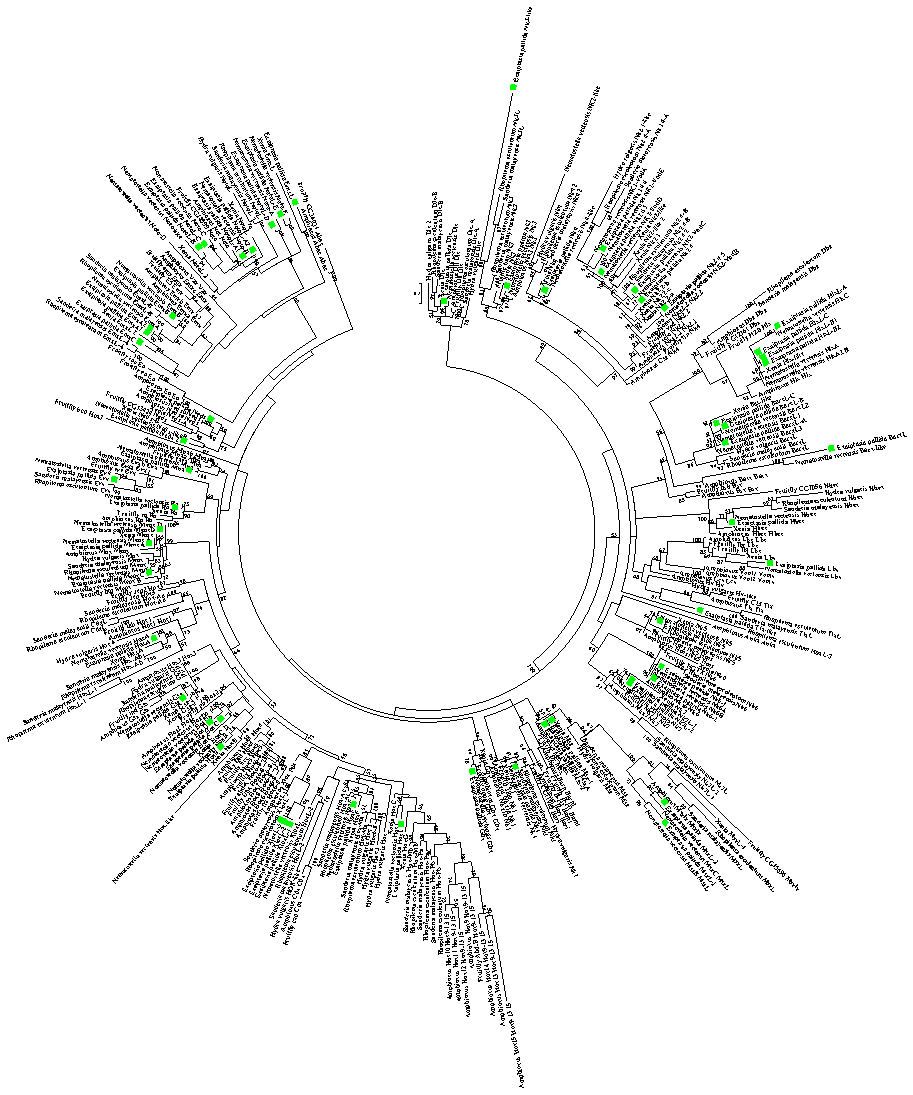


**Supplementary Figure 1. Maximum likelihood (ML) phylogenetic tree of ANTP-class homeobox genes under the LG+I+G4 model, with 1000 bootstrap replicates.** Green squares represent *Exaiptasia pallida*. Only bootstrap values higher than 50% are shown in the figure.


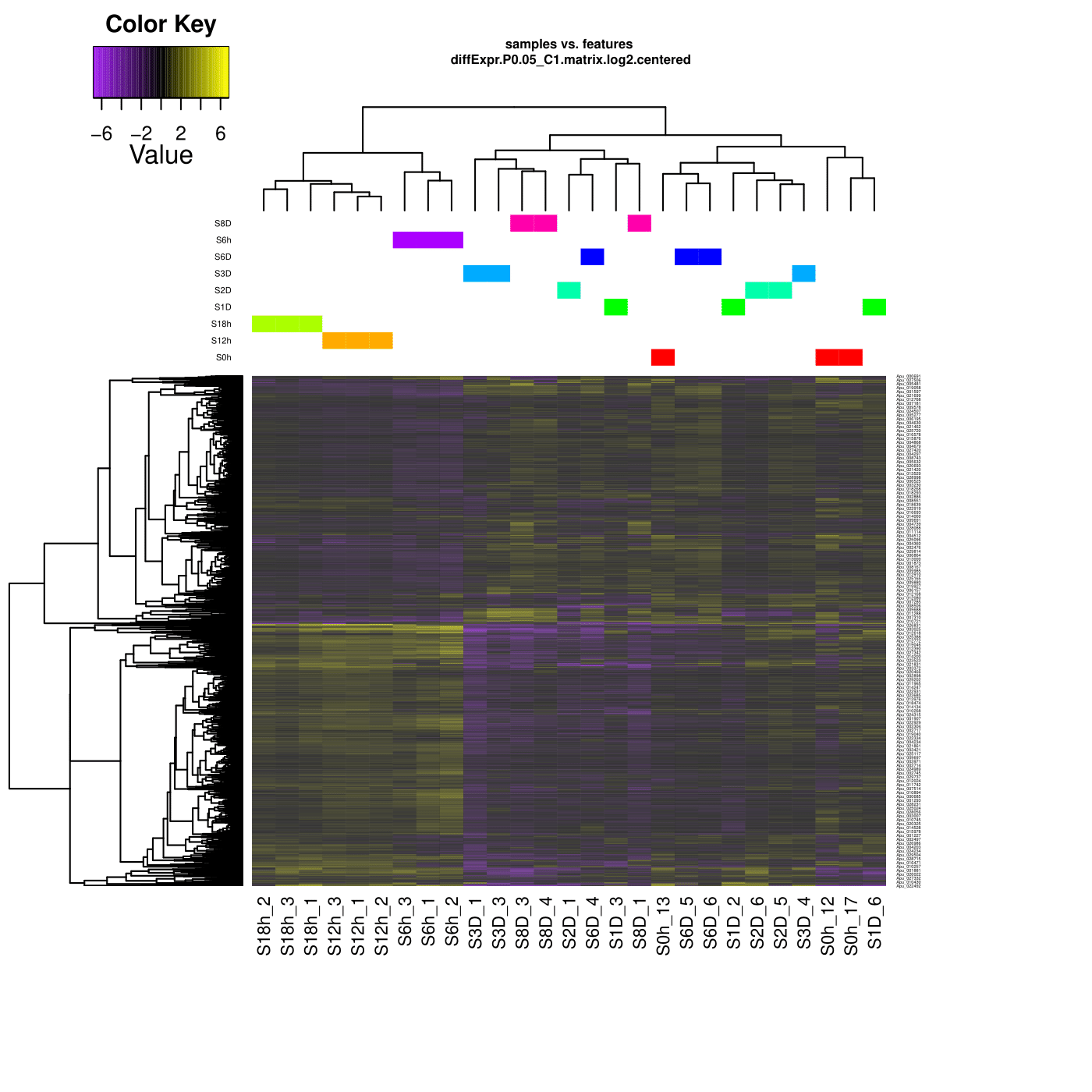


**Supplementary Figure 2. Heatmap showing the expression of genes during different time points of *Exaiptasia pallida* tentacle regeneration**. The shown genes have a minimum of 50 CPM in at least 3 samples. The cut-off values set were FDR = 0.05, logFC = 1 (2x).

**
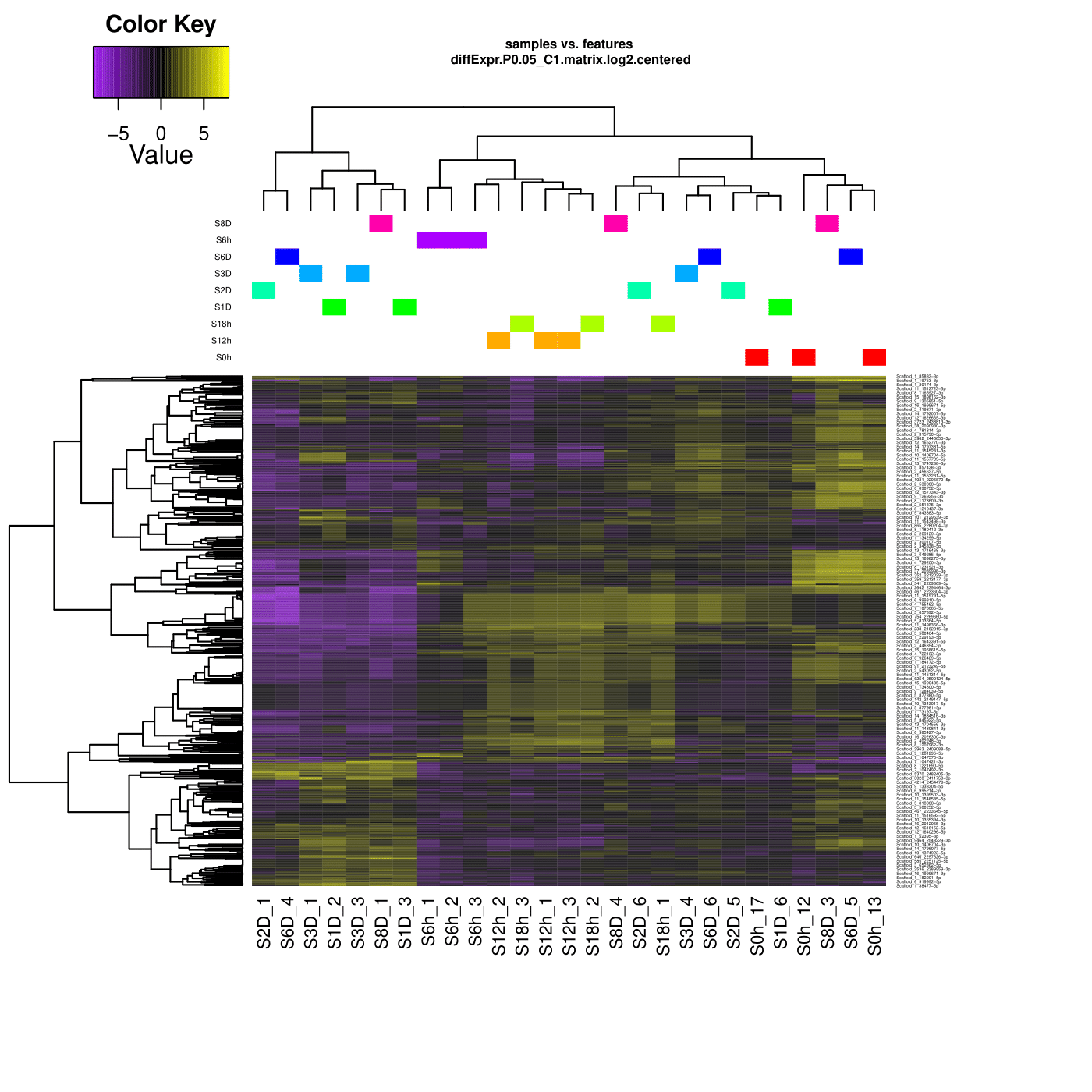
**

**Supplementary Figure 3. Heatmap showing the expression of miRNAs during different time points of *Exaiptasia pallida* tentacle regeneration.** The shown miRNAs have a minimum of 5 CPM in at least 3 samples. The cut-off values set were FDR = 0.05, logFC = 1 (2x).


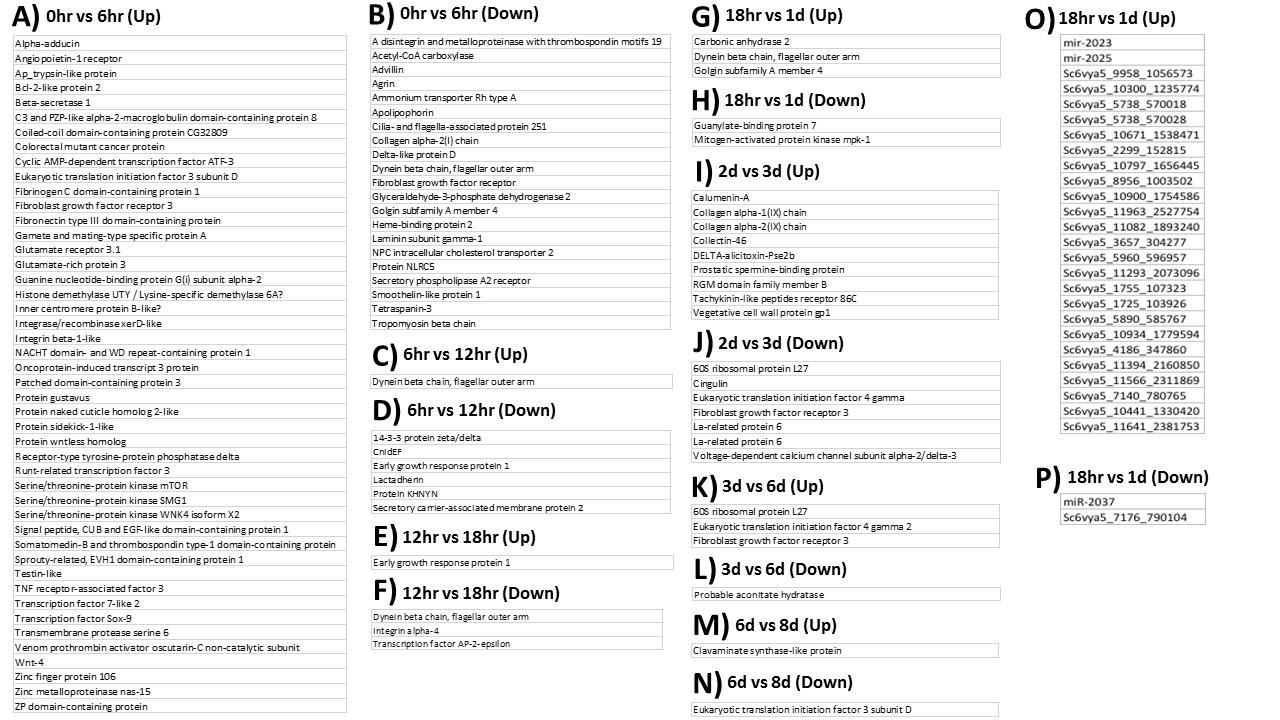


**Supplementary Figure 4. Lists of genes and microRNAs that show differential expression during different time points of *Exaiptasia pallida* tentacle regeneration.** A-N) Genes upregulated and downregulated during different time points. O-P) microRNAs upregulated and downregulated during different time points. Only those that fulfill the criteria of MirGeneDB (http://mirgenedb.org/information) are included in these lists.

**
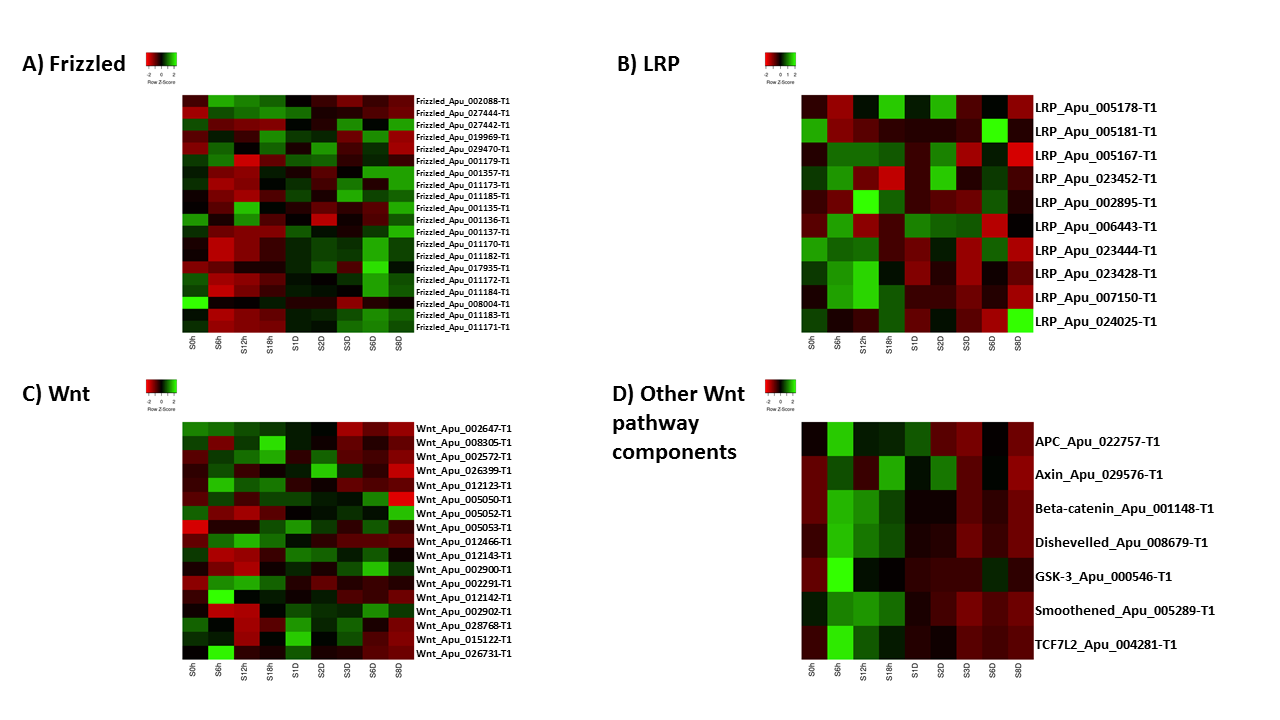
**

**Supplementary Figure 5.** **Heatmaps showing the expression of Wnt signaling pathway genes during different time points of *Exaiptasia pallida* tentacle regeneration.** A-D) Expression of A) *Frizzled*, B) *LRP*, C) *Wnt*, and D) other Wnt signaling pathway genes during *Exaiptasia pallida* tentacle regeneration.

**
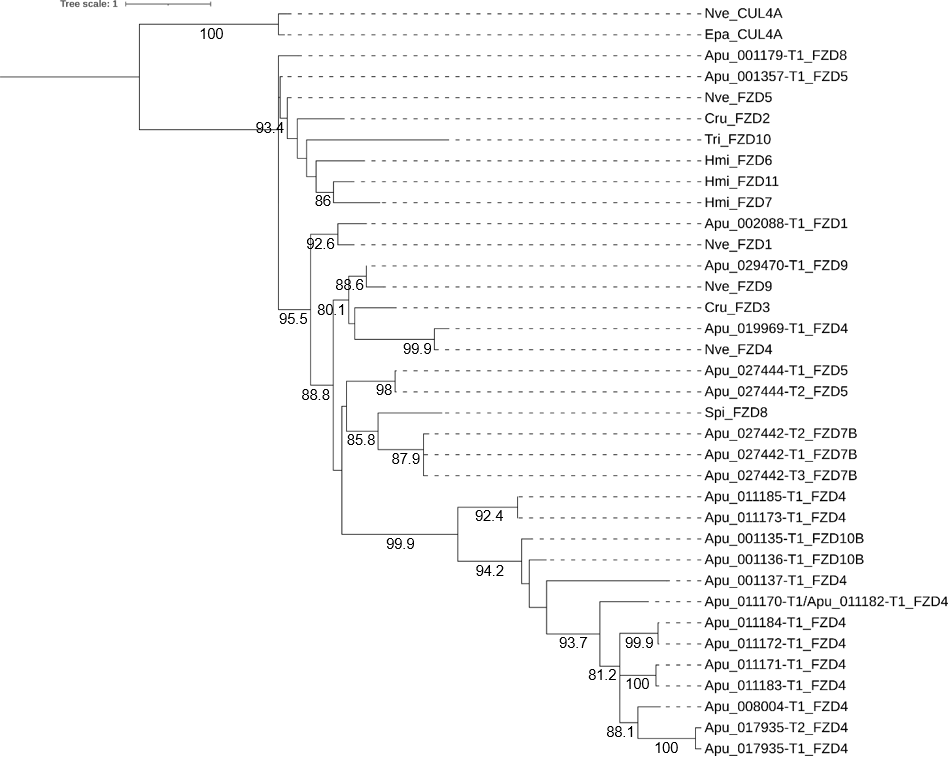
**

**Supplementary Figure 6. Maximum likelihood (ML) phylogenetic tree of Frizzled (FZD) genes in *Exaiptasia pallida* under the LG+I+G4 model, with 1000 bootstrap replicates.** The tree was rooted using Cullin-4A (CUL4A). Only bootstrap support values above 80% are indicated for clarity. The scale bar represents the expected number of nucleotide substitutions per site. Apu: *E. pallida* (from this study), Cru: *Corallium rubrum*, Epa: *E. pallida*, Hmi: *Hofstenia miamia*, Nve: *Nematostella vectensis*, Spi: *Stylophora pistillata*, Tri: *Trichoplax sp.*.


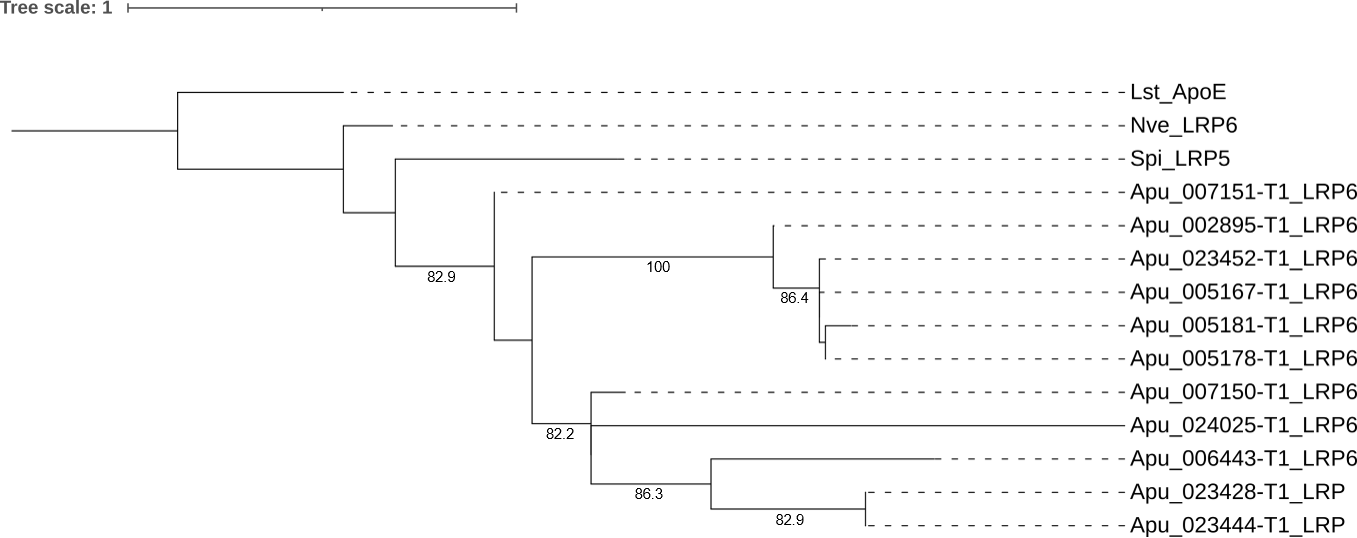


**Supplementary Figure 7. Maximum likelihood (ML) phylogenetic tree of LRP genes in *Exaiptasia pallida* under the FLU+G4 model, with 1000 bootstrap replicates.** The tree was rooted using Apolipoprotein E receptor (ApoE). Only bootstrap support values above 80% are indicated for clarity. The scale bar represents the expected number of nucleotide substitutions per site. Apu: *E. pallida* (from this study), Lst: *Lymnaea stagnalis*, Nve: *Nematostella vectensis*, Spi: *Stylophora pistillata*.


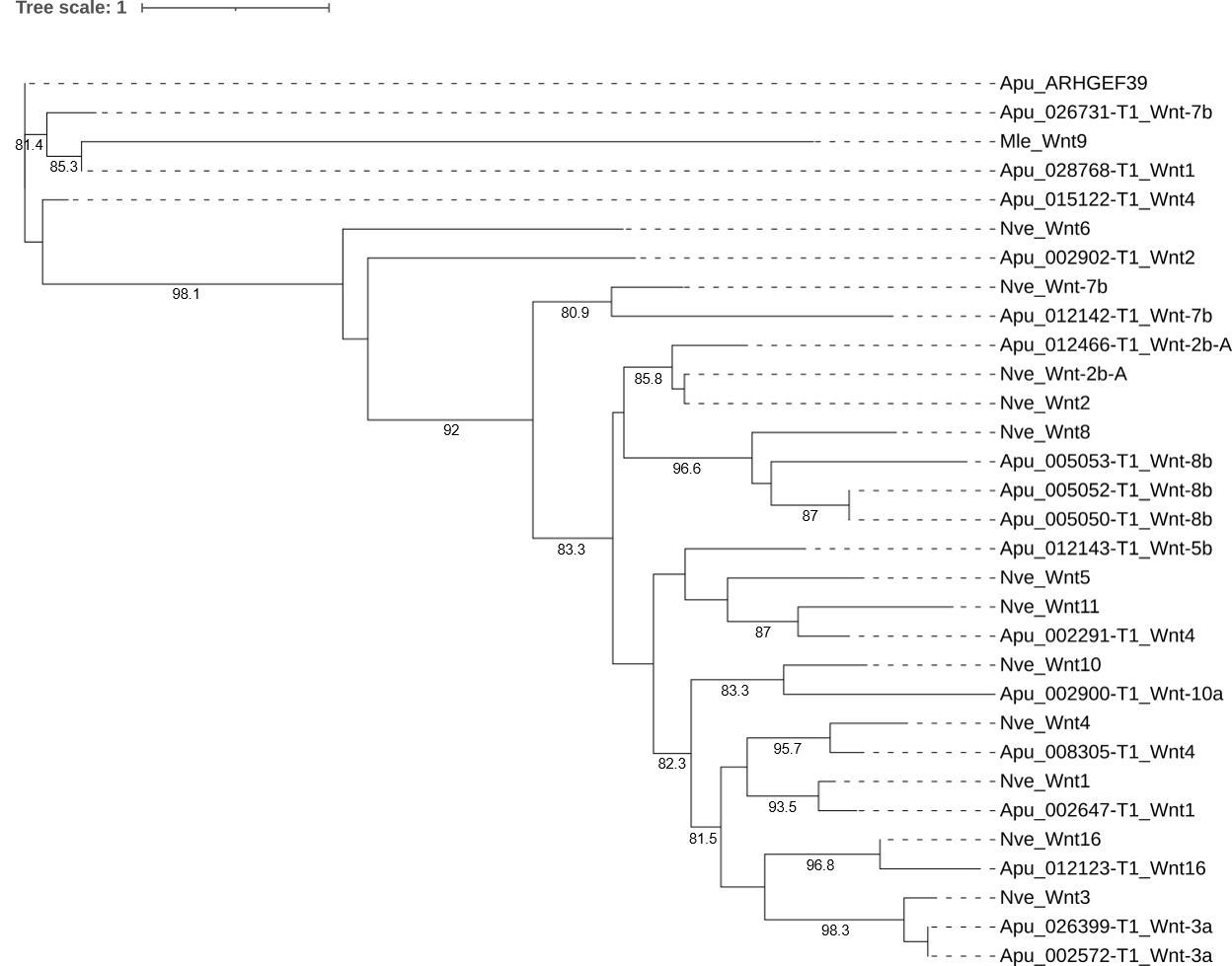


**Supplementary Figure 8. Maximum likelihood (ML) phylogenetic tree of Wnt genes in *Exaiptasia pallida* under the LG+I+G4 model, with 1000 bootstrap replicates.** The tree was rooted using Rho guanine nucleotide exchange factor 39 (ARHGEF39). Only bootstrap support values above 80% are indicated for clarity. The scale bar represents the expected number of nucleotide substitutions per site. Apu: *E. pallida* (from this study), Mle: *Mnemiopsis leidyi*, Nve: *Nematostella vectensis*.

**
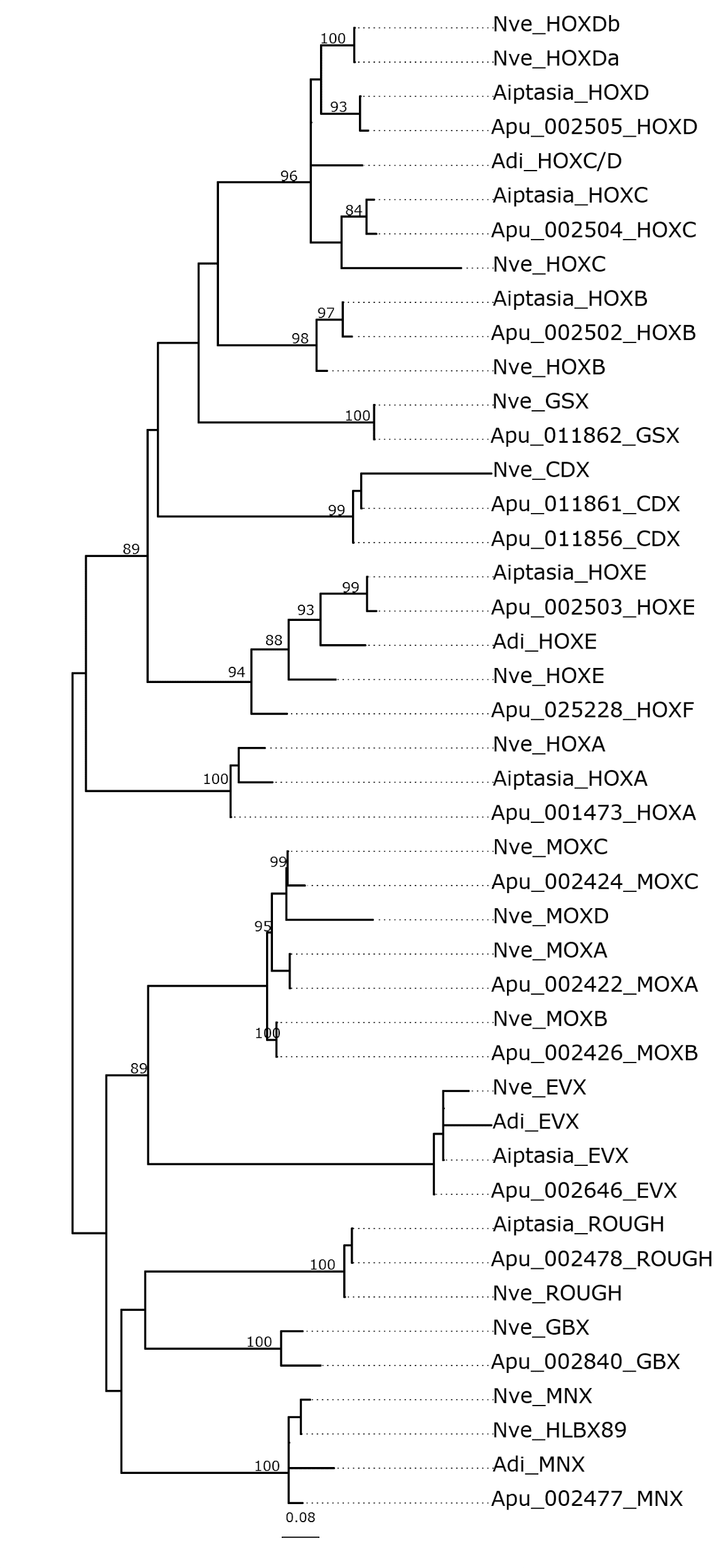
**

**Supplementary Figure 9. Maximum likelihood (ML) phylogenetic tree of Hox/ParaHox genes in *Exaiptasia pallida* under the LG+G4 model, with 1000 bootstrap replicates*.*** Only bootstrap support values above 80% are indicated for clarity. Adi: *Acropora digitifera*, Apu: *E. pallida* (from this study), Nve: *Nematostella vectensis*. The scale bar represents the expected number of nucleotide substitutions per site.

Signal peptide sequence

convertase site

potential mature neuropeptides

>Aip_PRGamide_Apu_014060-T1

MPFKKGFLLCLALELTITILCEARDVQRAYIGQRDFGDWYNNYEPYYEKQGSNKEPEEQDSFNFWDENDGPRGGRRSLDYQNGREINMYDGPRGGRSILENGPRGGRSLENGPRGGRSLSSNGPRGGRSLGYGPRGGRSIYDGPRGGRSVYYGPRGGRSIEYGPRGGRSLQYGPRGGRSLRNGPRGGRSLKRMQRAGPRGGRSLAQMGPRGGRSLSAKLRMKRNVLEDGVHGDRSVFNVFDGPRGGRSMEDEFGPRGGRSMDGPRGGRDESEGPRGGRDESEGPRGGRDEAEGPRGGRDEAEGPRGGRDEAEGPRGGRDEGEGPRGGRSITHDGPRGGRDVSTKNIDSHSRTKRVVSQNQLKASS

>Aip_GLWamide_Apu_002579-T1

MALKGQLCVILTTLLLIQCQGKSTKKENIEQHKAVQTSGAERTGSIAGELSEISEERREAEPPQFGLWGKRQVESPIEDPQFFDKKANSFGLWGKRGNGVGLWGRSADSWSKRQDSGLGLWGRSANPGNAVGLWGKRQRGGGRRGLDAKRYANPGDGVGLWGKRQHDFGLWGRSAEPGNPVGLWGRVADKRDEQKRQKSIGLWGRSADPQKIGLWGR

>Aip_HIRamide_Apu_022822-T1

MKNVLSLLSLAMMLSIVIATEEKKDAKEQPPFVDLTQPAFYHIRGKRVARQPPFVDLTQPAFYHIRGKRVAKQPPFAVDLTQPAFYHIRGKRQFPPNVDLTAPAYYHIRGKRVAQQPPFVDLTQPAYYHIRGKRDN

>Aip_RFamide_Trinity_GG_30402_c0_g2_i3.p1

MTTASYVTILVTLLFHILAINAKDTKREPEDDQPQFWKGRFARGAVPQYWQGRFSDPQFWKGRFADPQFWKGRFADPQFWKGRFADPQFWKGRFSDPQYWKGRFSDDDKRSNDPQYWKGRFSRSMKTPDDDLPQFWKGRFSRDSLPGRFGRELQGRFGREQGRFGREEQGRFGREEQGRFGREEQGRVG

**Supplementary Figure 10. Neuropeptide sequences identified in *Exaiptasia pallida.***

**
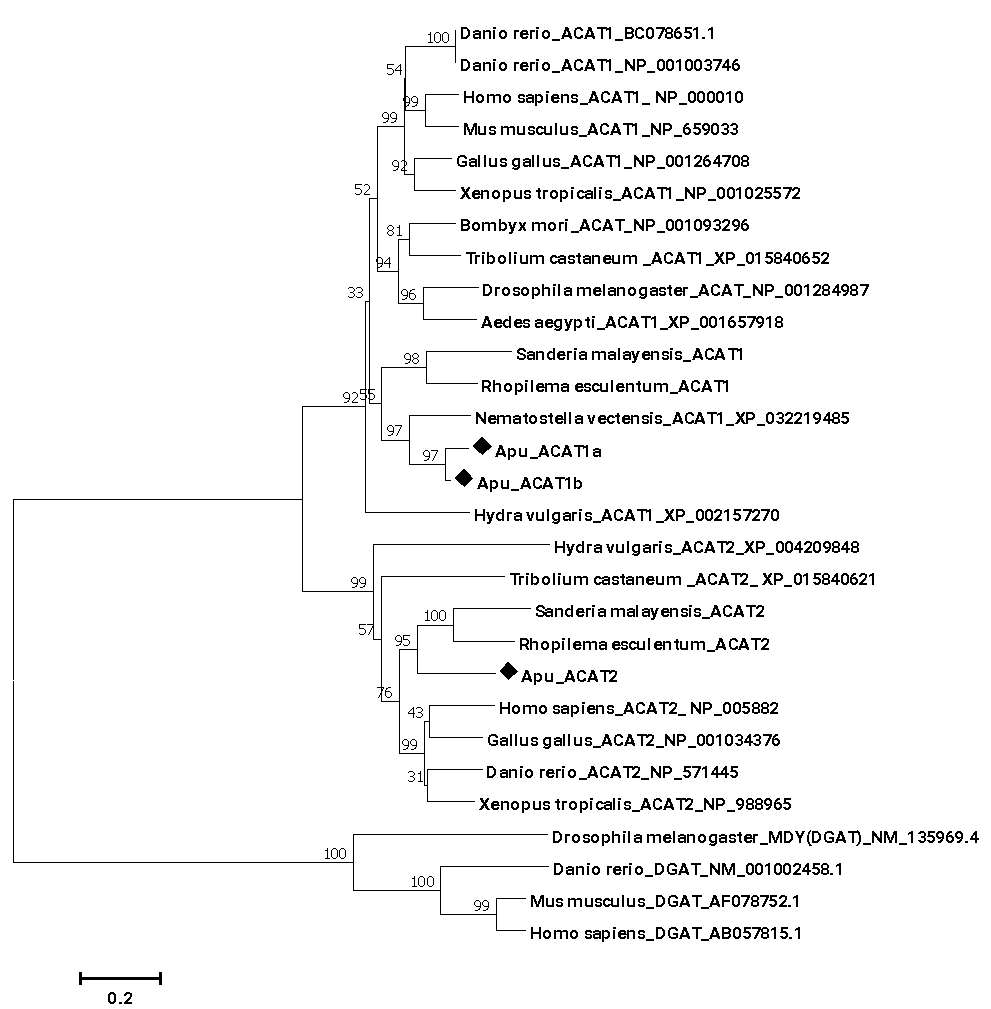
**

**Supplementary Figure 11. Phylogenetic tree of *acetyl-CoA C-acetyltransferase* (*ACAT*).** The tree was constructed with the Neighbor-Joining (NJ) method with 1000 bootstrap replicates and rooted using metazoan diglyceride acyltransferase (*DGAT*). The evolutionary distances were computed using the Poisson correction method and are in the units of the number of amino acid substitutions per site.

**
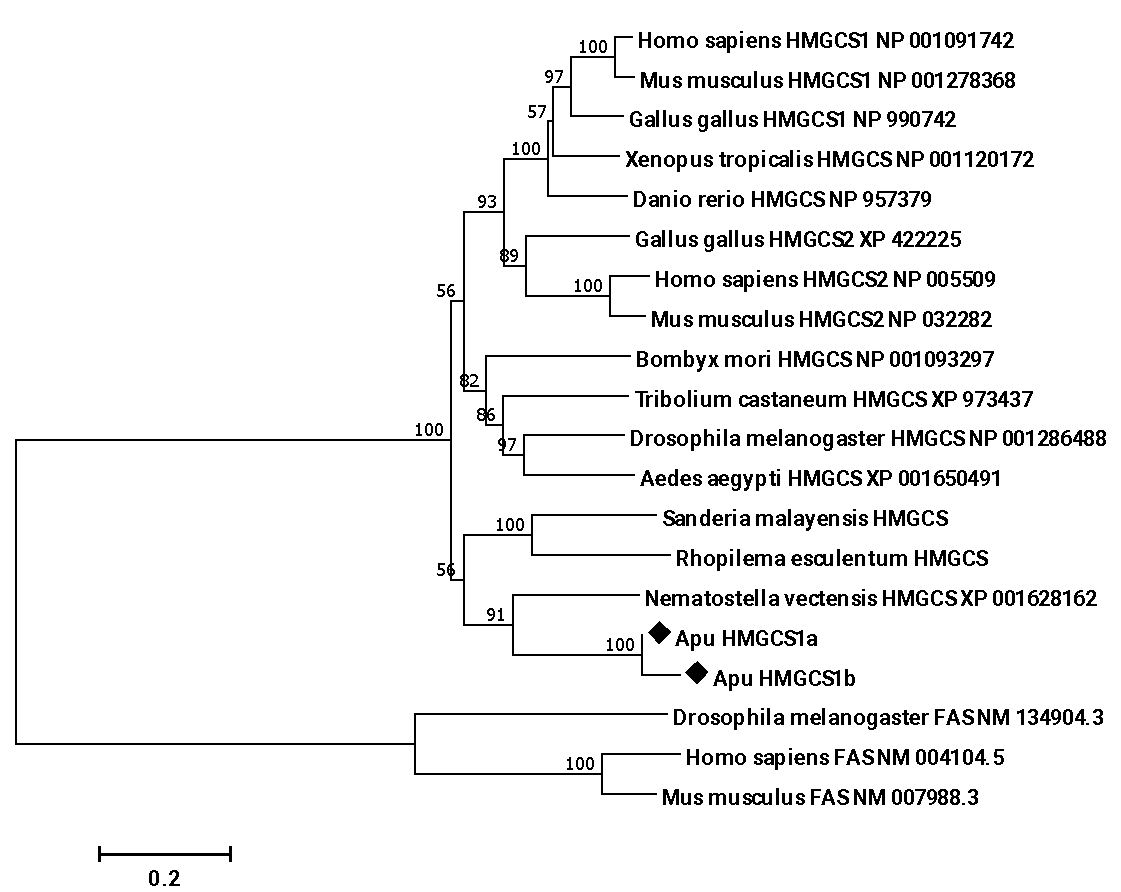
**

**Supplementary Figure 12. Phylogenetic tree of *hydroxymethylglutaryl-CoA synthase* (*HMGCS*).** The tree was constructed with the Neighbor-Joining (NJ) method with 1000 bootstrap replicates and rooted using metazoan fatty acid synthase (*FAS*). The evolutionary distances were computed using the Poisson correction method and are in the units of the number of amino acid substitutions per site.


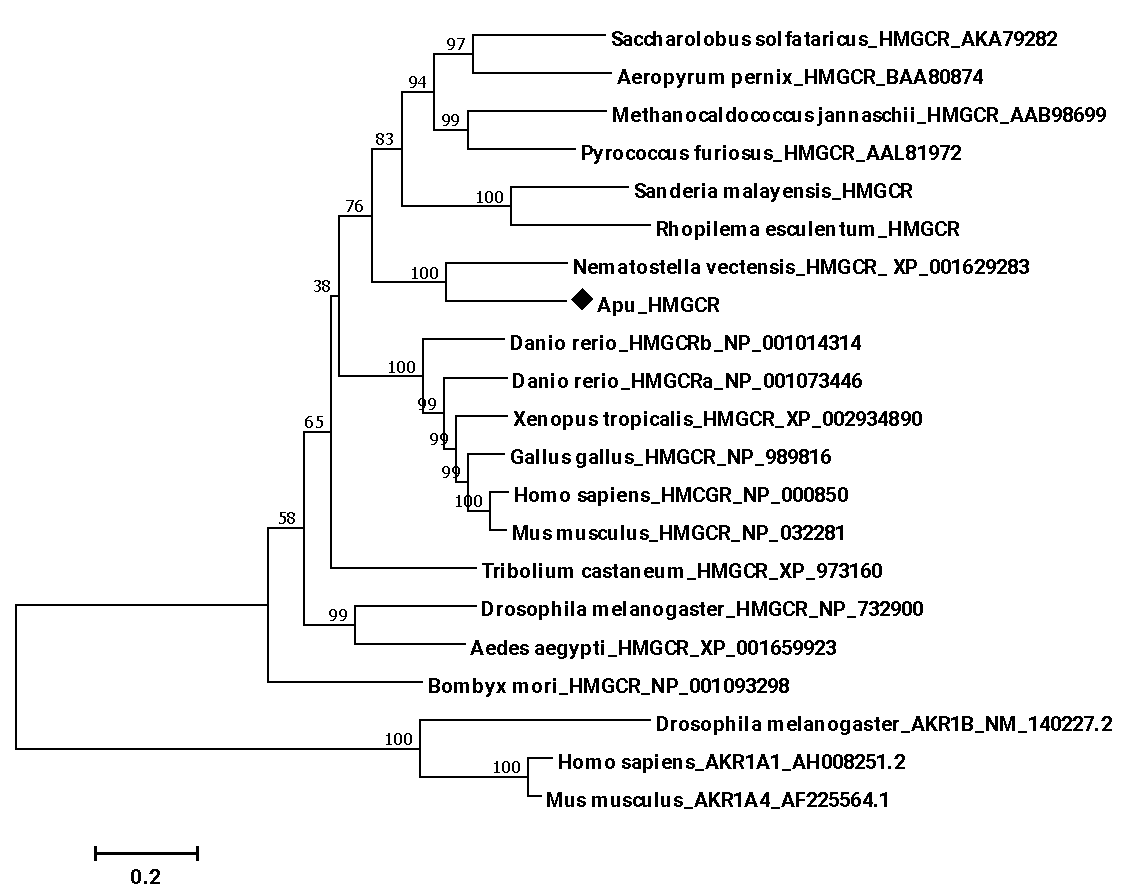


**Supplementary Figure 13. Phylogenetic tree of *hydroxymethylglutaryl-CoA reductase* (*HMGCR*).** The tree was constructed with the Neighbor-Joining (NJ) method with 1000 bootstrap replicates and rooted using metazoan aldo-keto reductase (*AKR1*). The evolutionary distances were computed using the Poisson correction method and are in the units of the number of amino acid substitutions per site.


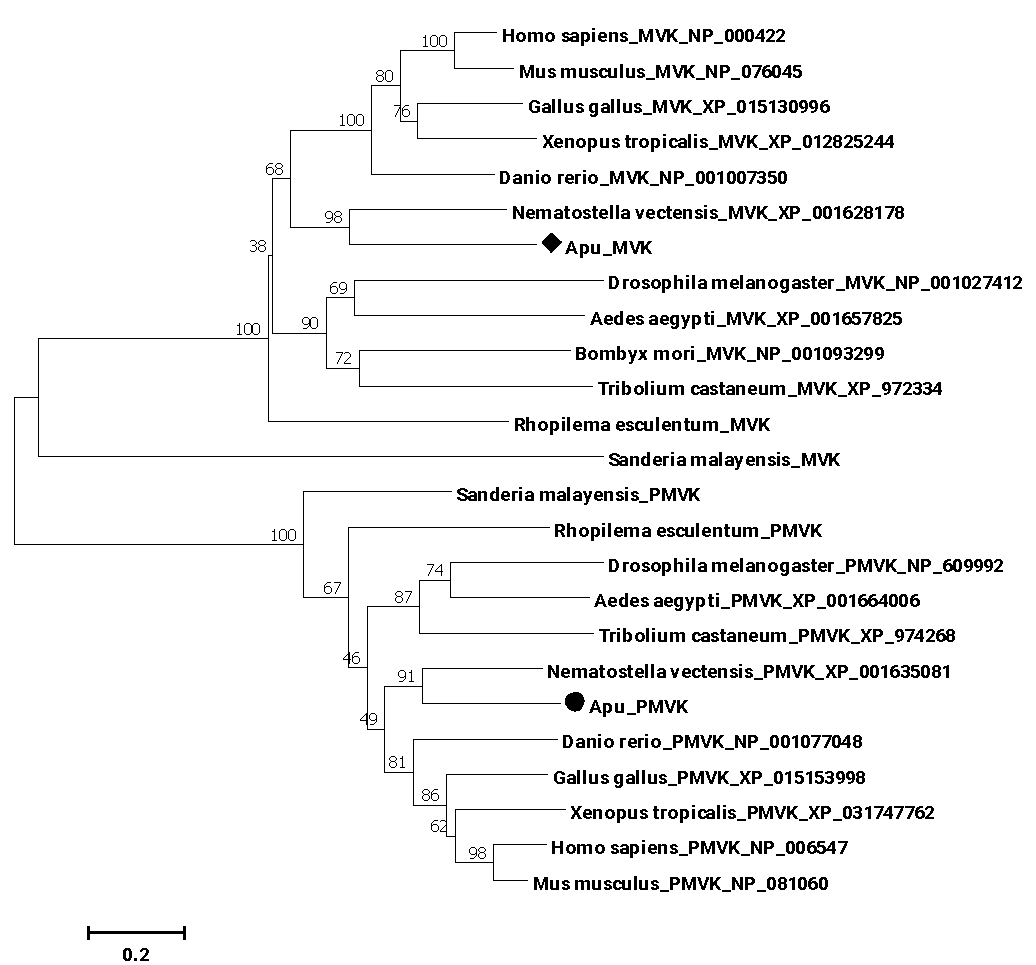


**Supplementary Figure 14. Phylogenetic tree of *mevalonate kinase* (MK) and *phosphomevalonate kinase* (*PMVK*).** The tree was constructed with the Neighbor-Joining (NJ) method with 1000 bootstrap replicates. The evolutionary distances were computed using the Poisson correction method and are in the units of the number of amino acid substitutions per site.


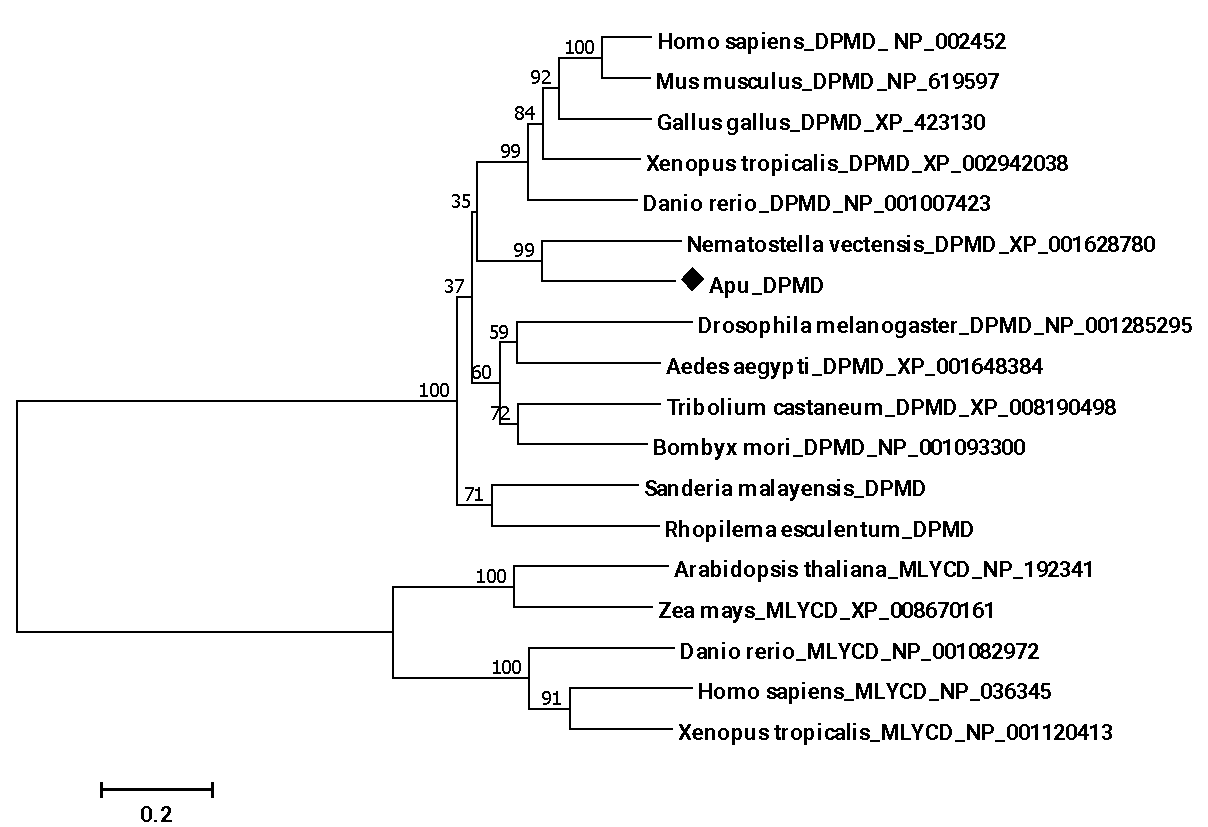


**Supplementary Figure 15. Phylogenetic tree of *diphosphomevalonate decarboxylase* (*DPMD*).** The tree was constructed with the Neighbor-Joining (NJ) method with 1000 bootstrap replicates and rooted using malonyl-CoA decarboxylase (*MLYCD*). The evolutionary distances were computed using the Poisson correction method and are in the units of the number of amino acid substitutions per site.
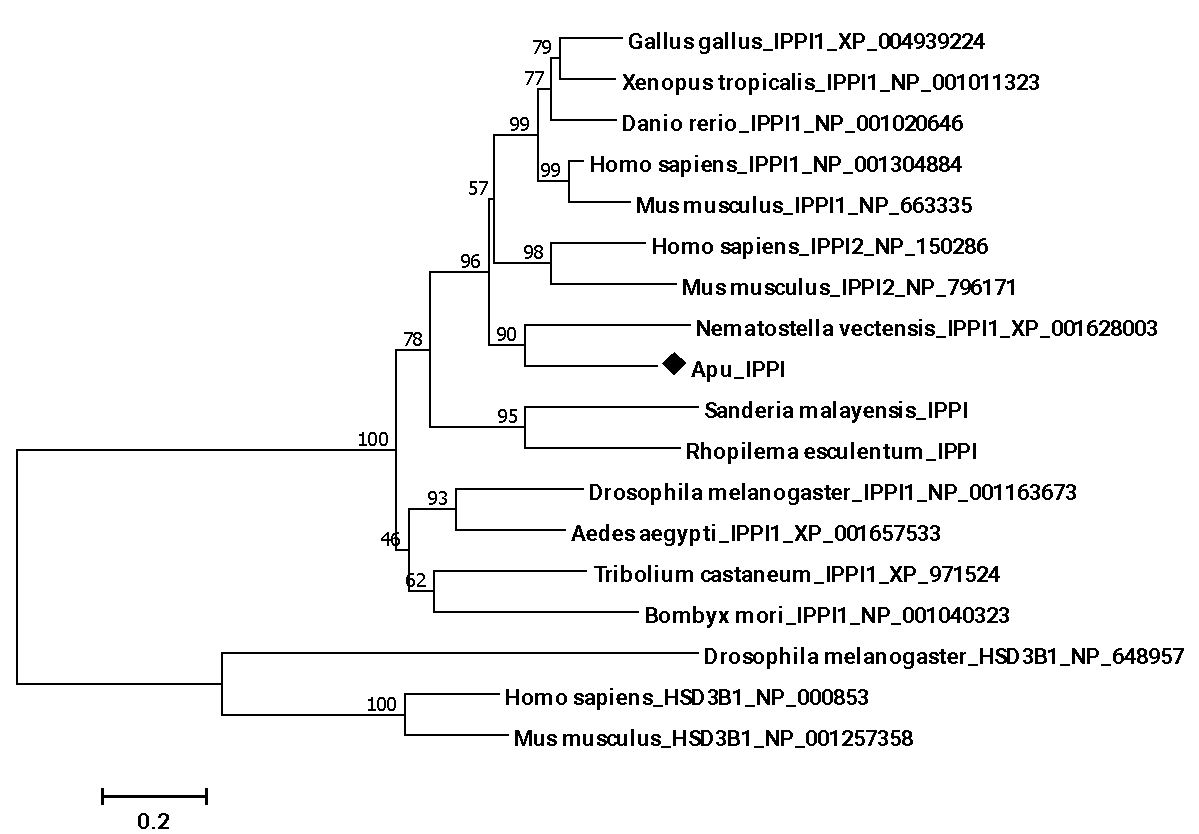


**Supplementary Figure 16. Phylogenetic tree of *isopentenyl-diphosphate delta-isomerase* (*IPPI*).** The tree was constructed with the Neighbor-Joining (NJ) method with 1000 bootstrap replicates and rooted using metazoan hydroxy-delta-5-steroid dehydrogenase, 3 beta- and steroid delta-isomerase 1 (*HSD3B1*). The evolutionary distances were computed using the Poisson correction method and are in the units of the number of amino acid substitutions per site.


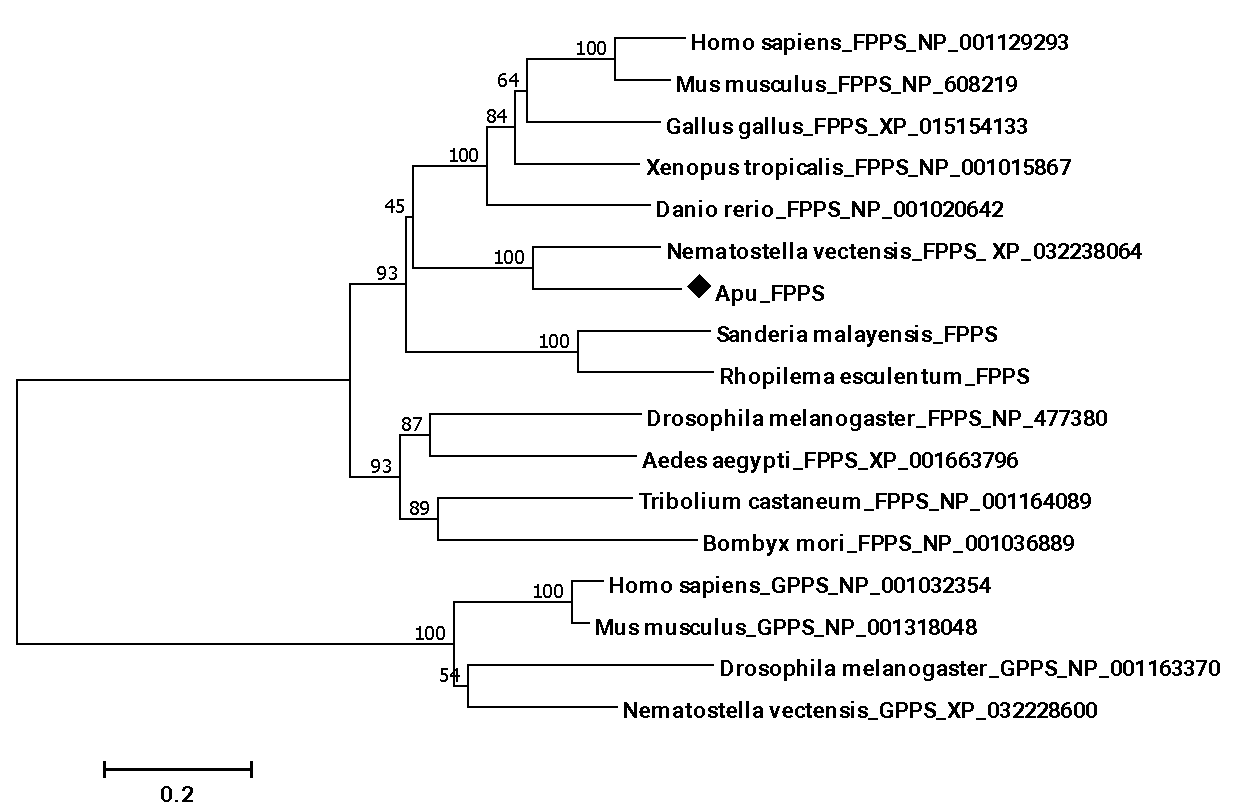


**Supplementary Figure 17. Phylogenetic tree of *farnesyl pyrophosphate synthase* (*FPPS*).** The tree was constructed with the Neighbor-Joining (NJ) method with 1000 bootstrap replicates and rooted using metazoan geranyl diphosphate synthase (*GPPS*). The evolutionary distances were computed using the Poisson correction method and are in the units of the number of amino acid substitutions per site.


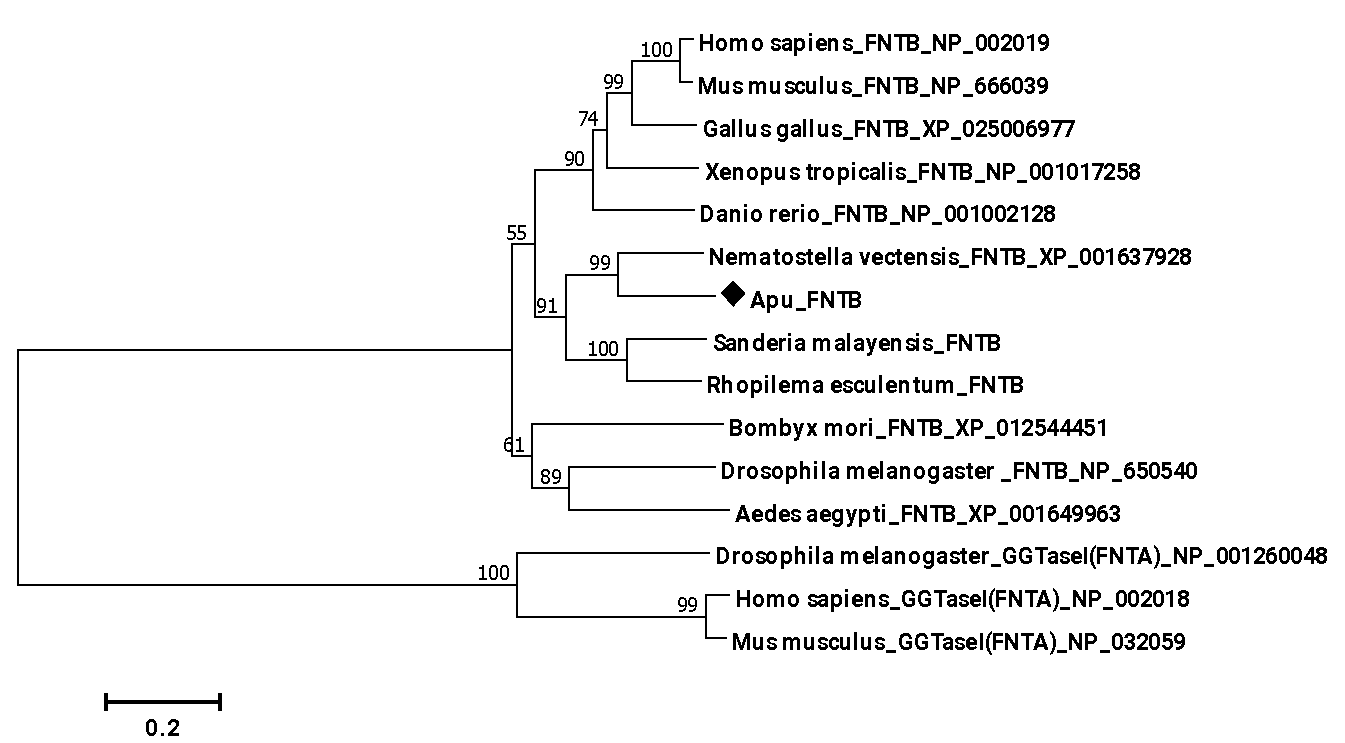


**Supplementary Figure 18. Phylogenetic tree of *farnesyltransferase beta* (*FNTB*).** The tree was constructed with the Neighbor-Joining (NJ) method with 1000 bootstrap replicates and rooted using metazoan geranylgeranyltransferase (*FNTA*). The evolutionary distances were computed using the Poisson correction method and are in the units of the number of amino acid substitutions per site.


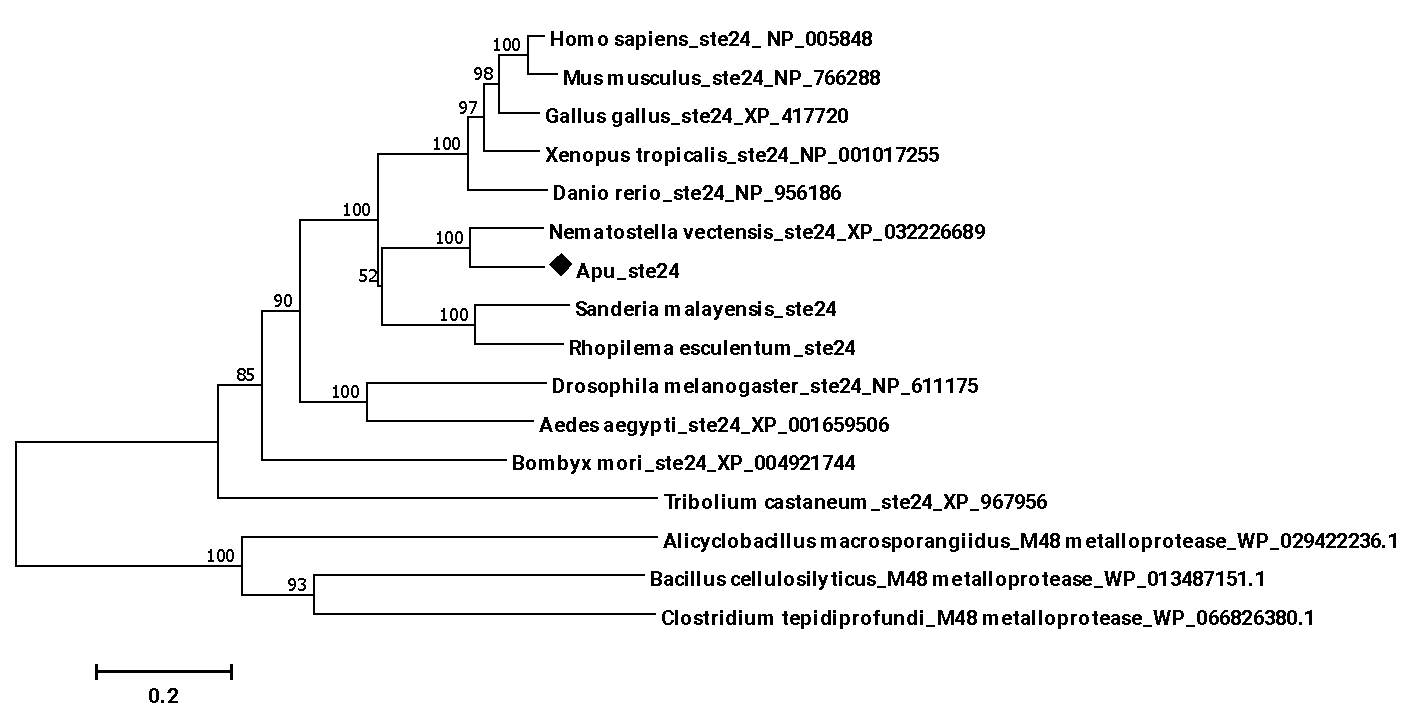


**Supplementary Figure 19. Phylogenetic tree of *ste24 endopeptidase* (*ste24*).** The tree was constructed with the Neighbor-Joining (NJ) method with 1000 bootstrap replicates and rooted using protozoan M48 metalloprotease. The evolutionary distances were computed using the Poisson correction method and are in the units of the number of amino acid substitutions per site.


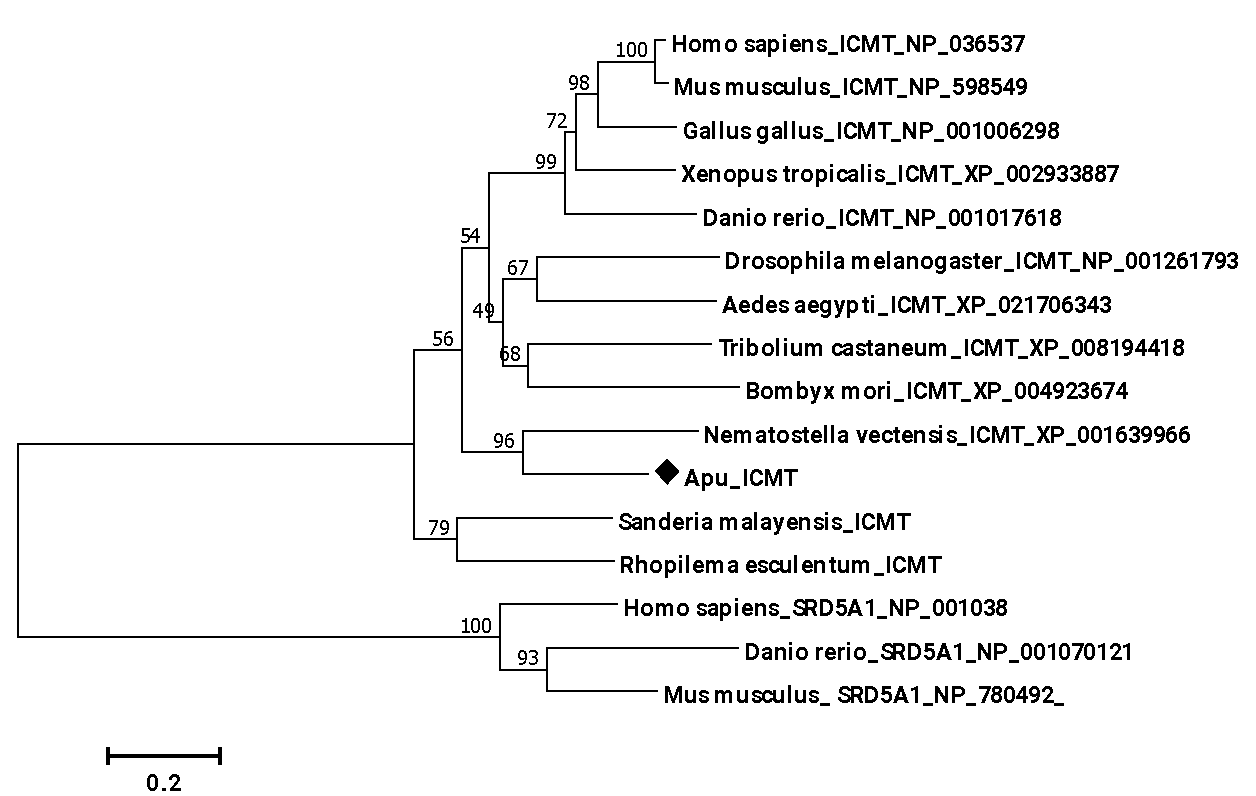


**Supplementary Figure 20. Phylogenetic tree of *protein-S-isoprenylcysteine O-methyltransferase* (*ICMT*).** The tree was constructed with the Neighbor-Joining (NJ) method with 1000 bootstrap replicates and rooted using metazoan steroid 5 alpha-reductase 1 (*SRD5A1*). The evolutionary distances were computed using the Poisson correction method and are in the units of the number of amino acid substitutions per site.


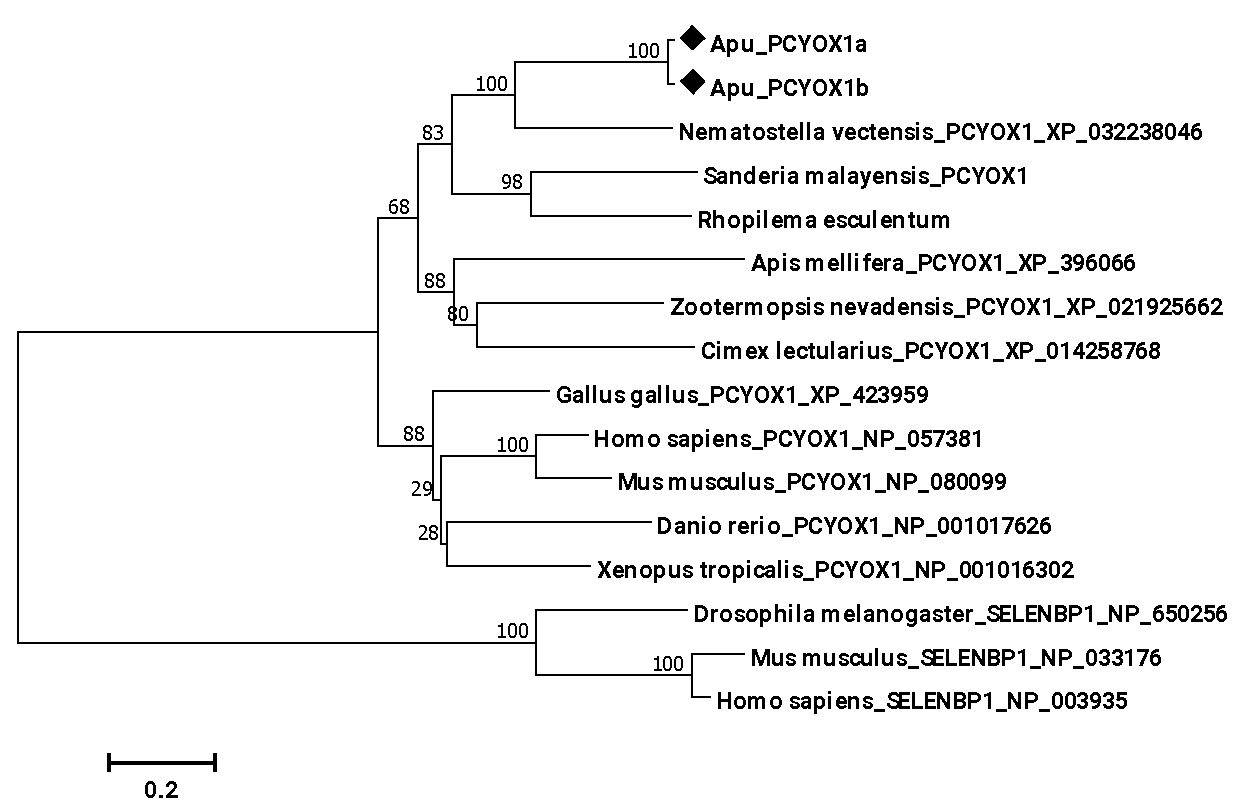


**Supplementary Figure 21. Phylogenetic tree of *prenylcysteine oxidase* (*PCYOX1*).** The tree was constructed with the Neighbor-Joining (NJ) method with 1000 bootstrap replicates and rooted using metazoan selenium binding protein 1 (*SELENBP1*). The evolutionary distances were computed using the Poisson correction method and are in the units of the number of amino acid substitutions per site.


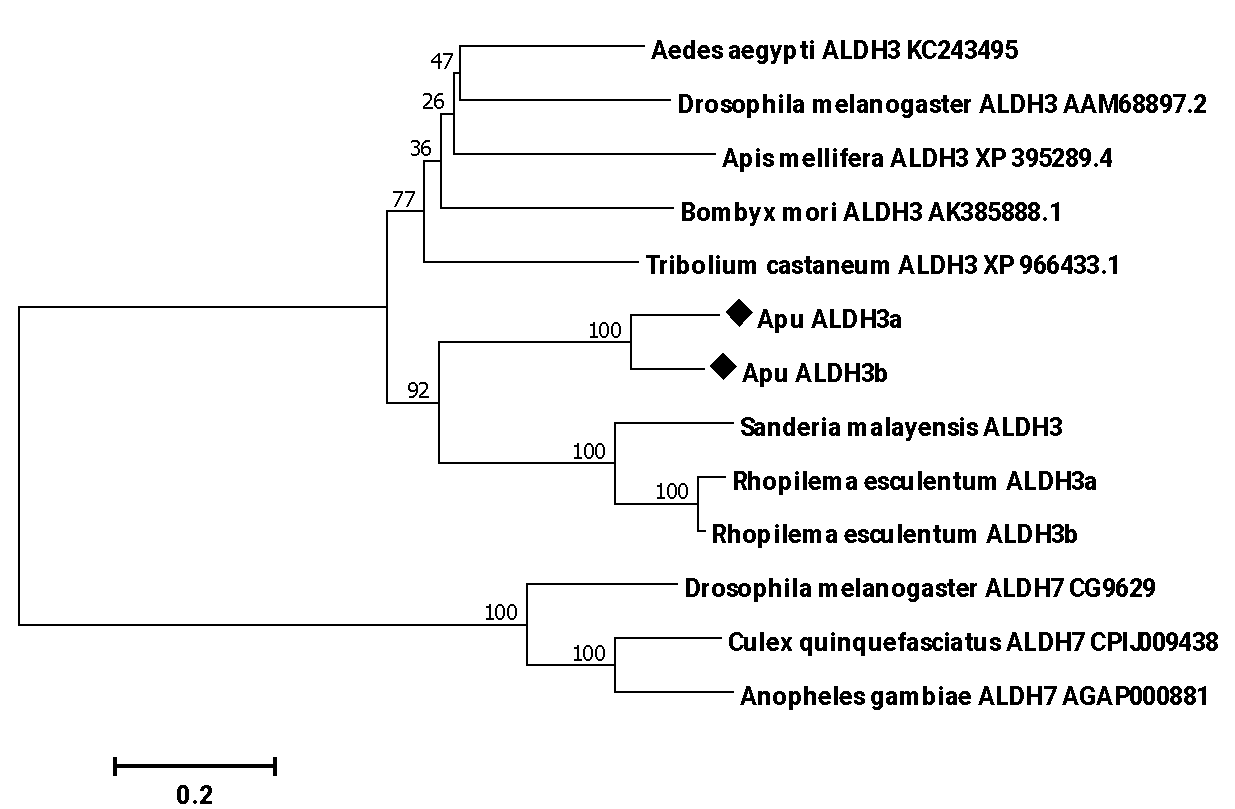


**Supplementary Figure 22. Phylogenetic tree of *aldehyde dehydrogenase 3* (*ALDH3*).** The tree was constructed with the Neighbor-Joining (NJ) method with 1000 bootstrap replicates and rooted using arthropod aldehyde dehydrogenase 7 (*ALDH7*). The evolutionary distances were computed using the Poisson correction method and are in the units of the number of amino acid substitutions per site.

**
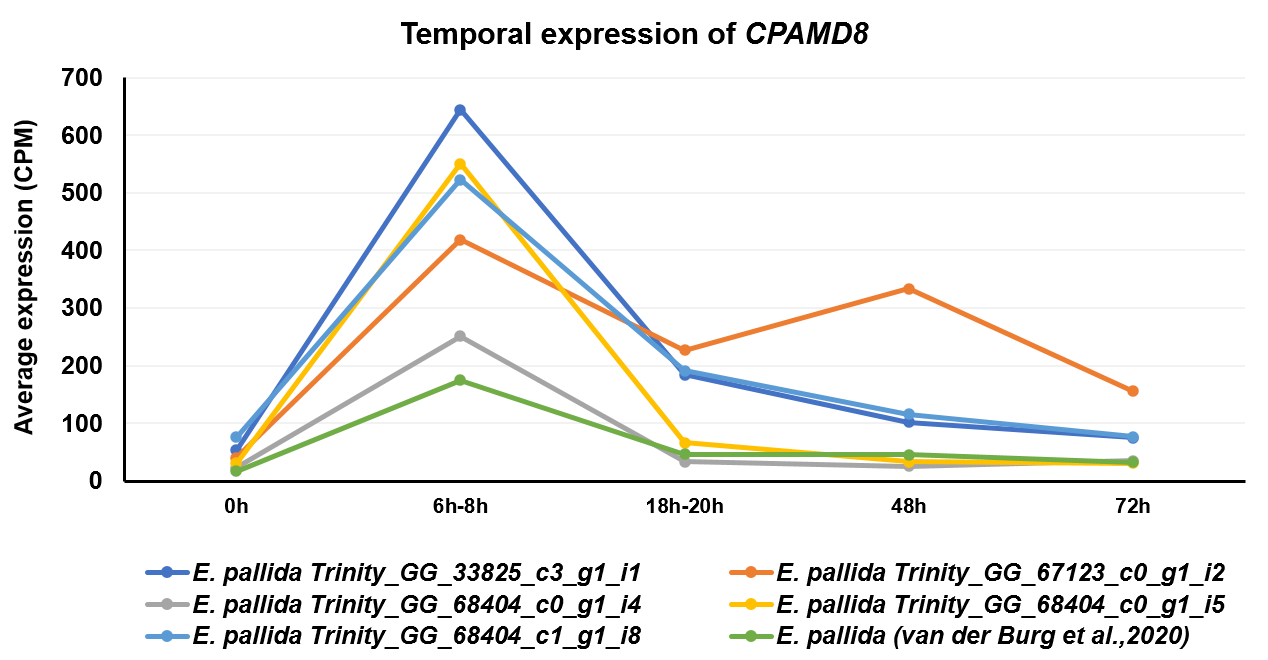
**

**Supplementary Figure 23. Temporal expression of *C3 and PZP-like alpha-2-macroglobulin domain-containing protein 8* during different time points in *Exaiptasia pallida* (this study) and *E. pallida* (van der Burg et al., 2020).**

**
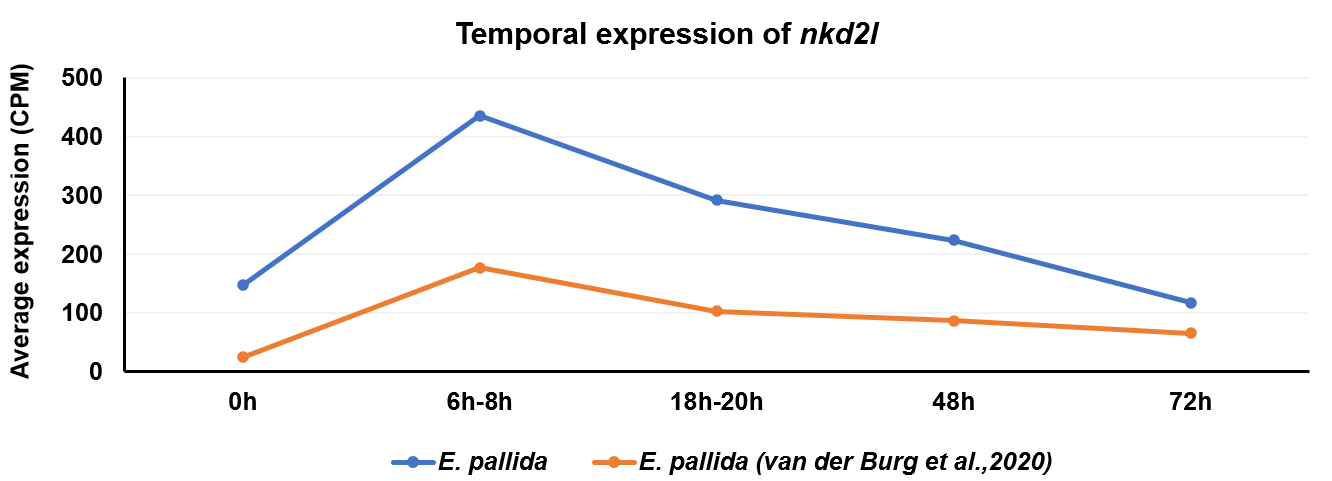
**

**Supplementary Figure 24. Temporal expression of *protein naked cuticle homolog 2-like* during different time points in *Exaiptasia pallida* (this study) and *E. pallida* (van der Burg et al., 2020).**

**
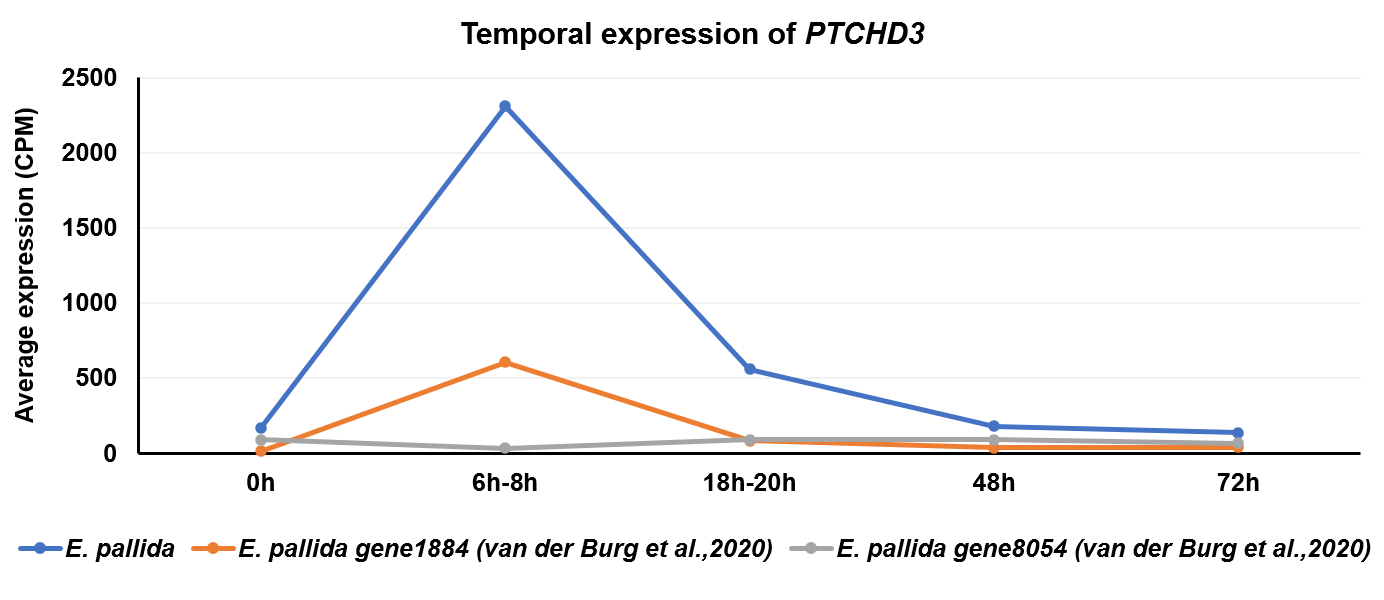
**

**Supplementary Figure 25. Temporal expression of *patched domain-containing protein 3* during different time points in *Exaiptasia pallida* (this study) and *E. pallida* (van der Burg et al., 2020).**


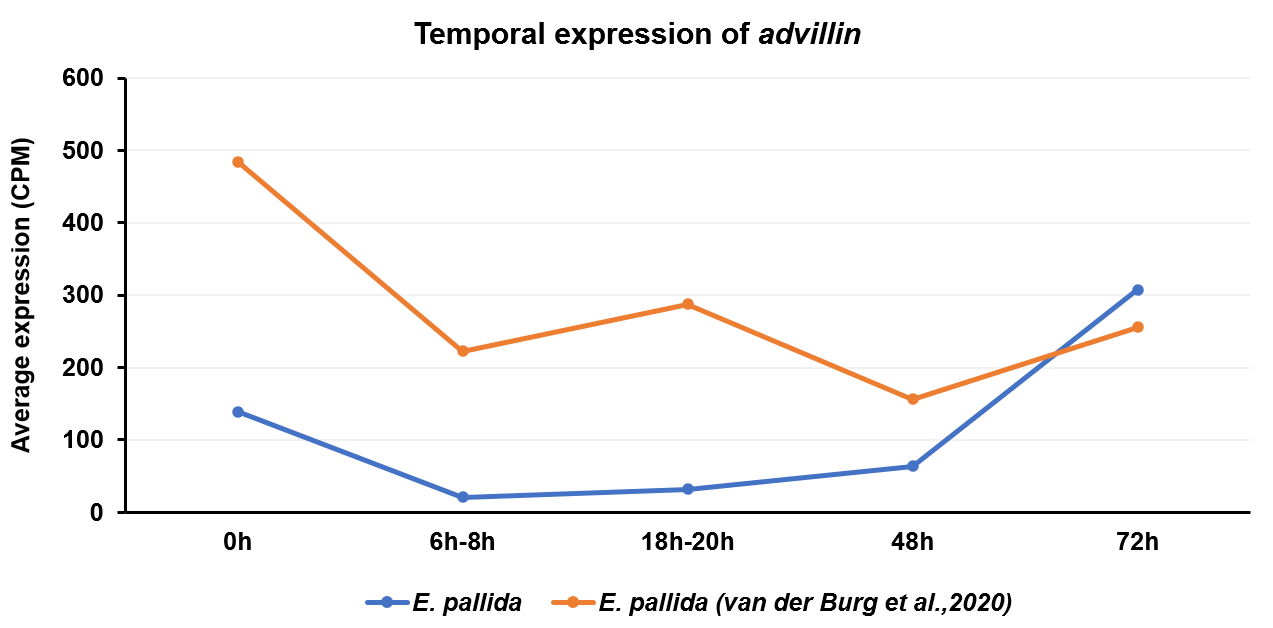


**Supplementary Figure 26. Temporal expression of *advillin* during different time points in *Exaiptasia pallida* (this study) and *E. pallida* (van der Burg et al., 2020).**


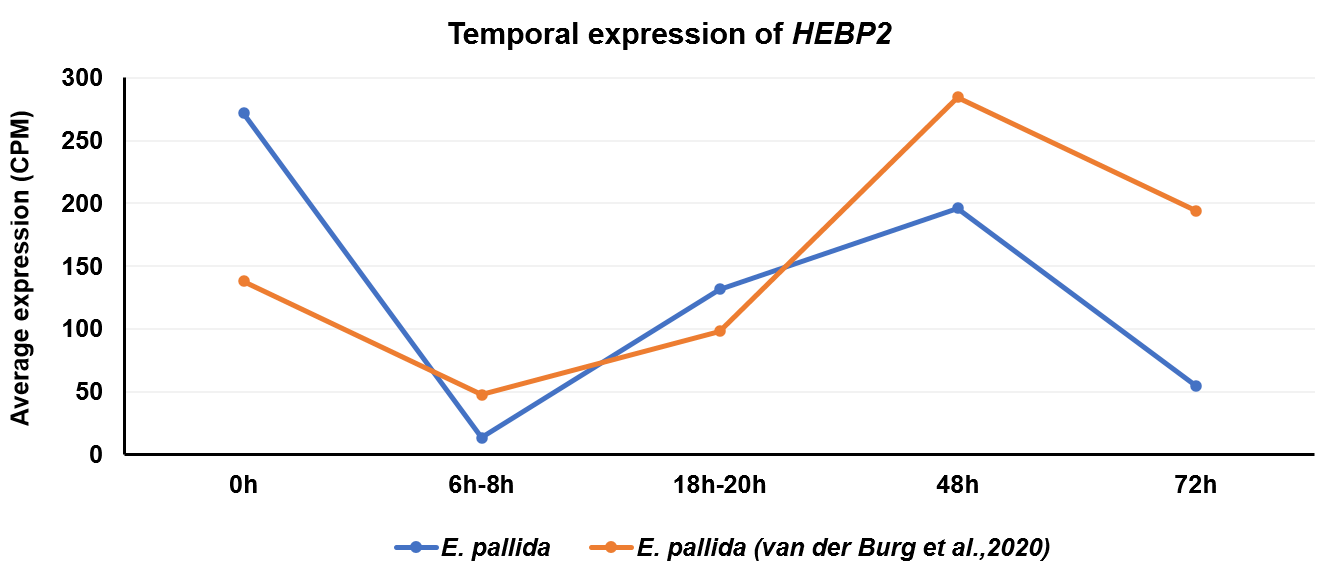


**Supplementary Figure 27. Temporal expression of *heme-binding protein 2* during different time points in *Exaiptasia pallida* (this study) and *E. pallida* (van der Burg et al., 2020).**


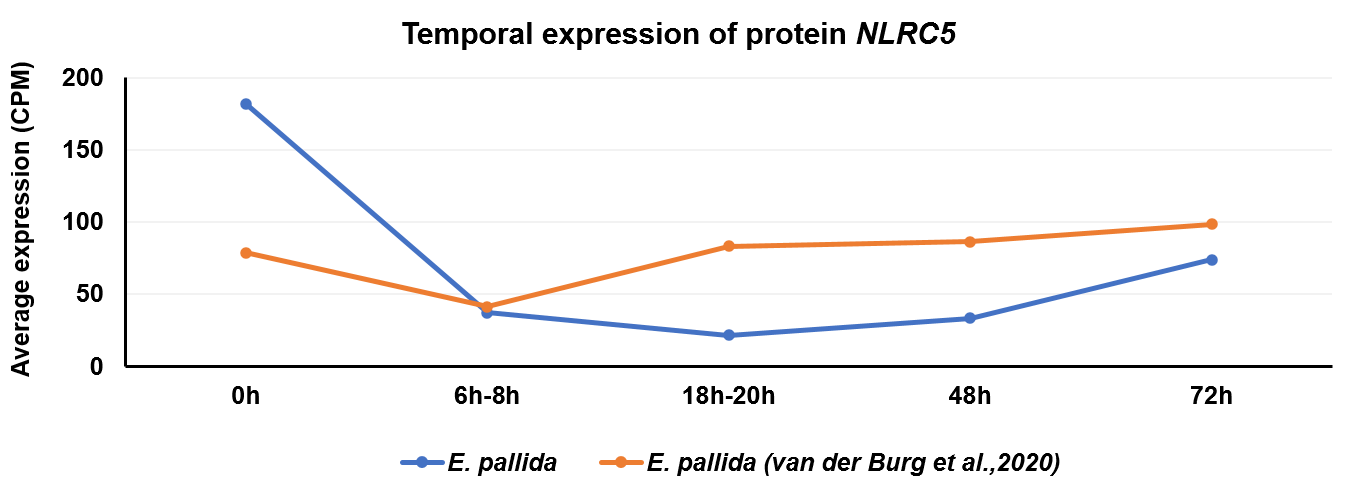


**Supplementary Figure 28. Temporal expression of protein *NLRC5* during different time points in *Exaiptasia pallida* (this study) and *E. pallida* (van der Burg et al., 2020).**


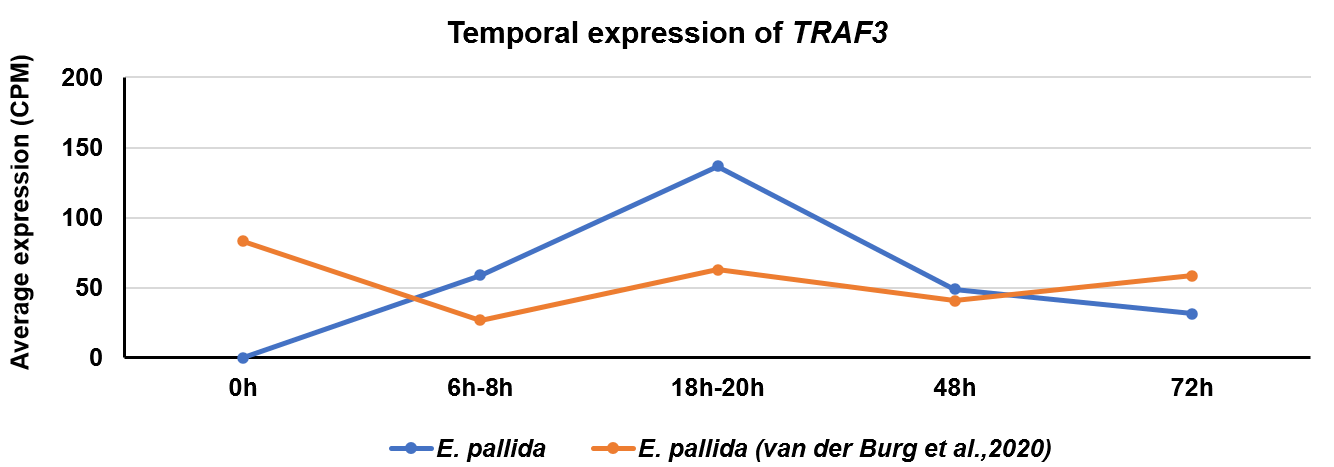


**Supplementary Figure 29. Temporal expression of *TNF receptor-associated factor 3* during different time points in *Exaiptasia pallida* (this study) and *E. pallida* (van der Burg et al., 2020).**


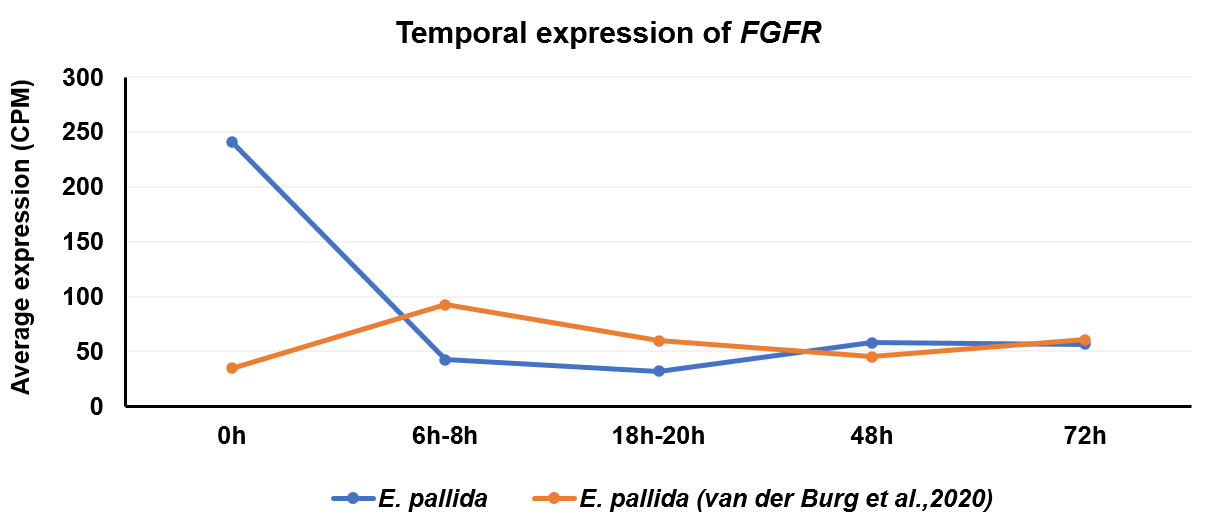


**Supplementary Figure 30. Temporal expression of *fibroblast growth factor receptor*during different time points in *Exaiptasia pallida* (this study) and *E. pallida* (van der Burg et al., 2020).**


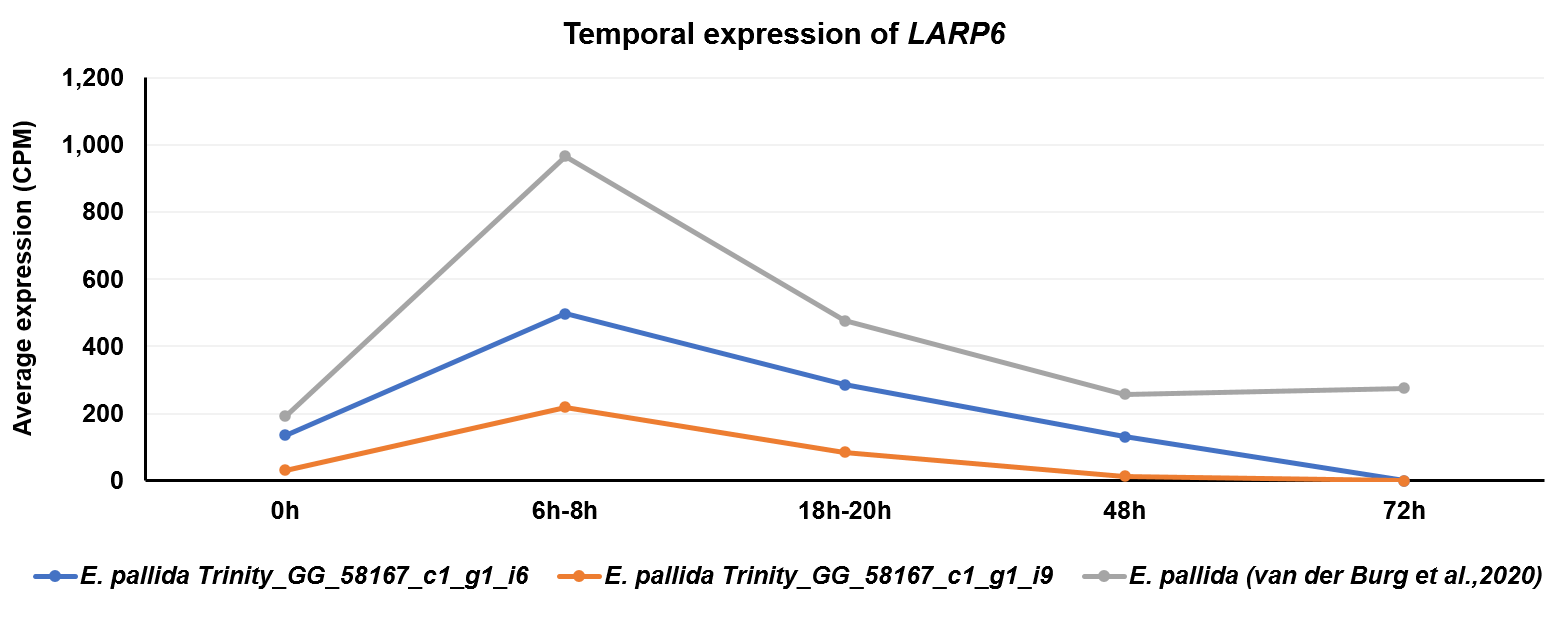


**Supplementary Figure 31. Temporal expression of *la-related protein 6* during different time points in *Exaiptasia pallida* (this study) and *E. pallida* (van der Burg et al., 2020).**
